# *Haloferax volcanii*—a model archaeon for studying DNA replication and repair

**DOI:** 10.1098/rsob.200293

**Published:** 2020-12-02

**Authors:** Patricia Pérez-Arnaiz, Ambika Dattani, Victoria Smith, Thorsten Allers

**Affiliations:** School of Life Sciences, University of Nottingham, Queen's Medical Centre, Nottingham, UK

**Keywords:** Archaea, *Haloferax volcanii*, DNA replication, DNA repair, homologous recombination

## Abstract

The tree of life shows the relationship between all organisms based on their common ancestry. Until 1977, it comprised two major branches: prokaryotes and eukaryotes. Work by Carl Woese and other microbiologists led to the recategorization of prokaryotes and the proposal of three primary domains: Eukarya, Bacteria and Archaea. Microbiological, genetic and biochemical techniques were then needed to study the third domain of life. *Haloferax volcanii*, a halophilic species belonging to the phylum Euryarchaeota, has provided many useful tools to study Archaea, including easy culturing methods, genetic manipulation and phenotypic screening. This review will focus on DNA replication and DNA repair pathways in *H. volcanii*, how this work has advanced our knowledge of archaeal cellular biology, and how it may deepen our understanding of bacterial and eukaryotic processes.

## 1. Haloferax volcanii

Pioneering work in the 1970s by Carl Woese and other microbiologists led to a profound reorganization of the tree of life. Woese's discovery of Archaea was initially based on small-subunit ribosomal RNA sequences [1], but was soon consolidated by work from Wolfram Zillig on RNA polymerase [2] and Otto Kandler on cell membranes [3]. Eventually, archaea took their place as members of a *bona fide* domain, alongside Eukarya and Bacteria [4]. Archaea share morphological features with bacteria—both are prokaryotic cells—but they show dramatic differences at the enzymatic level. The information processing machinery found in archaea, which includes the enzymes involved in DNA replication, is strikingly similar to that of eukaryotes. In the decades since their discovery, archaea have been shown to be neither ‘exotic bacteria’ nor ‘simplified eukaryotes’; instead, they display a mosaic of eukaryotic, bacterial and uniquely archaeal features. Furthermore, the recent discovery of Asgard archaea has provided support for a two-domain tree of life, where eukaryotes emerge from within the archaeal clade [5–7]. Thus, further study of archaea is needed to deepen our understanding of fundamental processes such as DNA replication and repair, and to shed light on our evolutionary history.

One of the model archaeal species is *Haloferax volcanii*, which is a member of the phylum Euryarchaeota. It is a halophile with disc-shaped cells and grows optimally at 45°C in 1.7–2.5 M NaCl, similar to the conditions found in the Dead Sea where it was first isolated in 1975 [8]. *Haloferax volcanii* cells do not possess a rigid cell wall but are instead surrounded by a glycoprotein surface (S-) layer, which can be a target for glycosylation [9]. *Haloferax volcanii* use a ‘salt-in’ mechanism to deal with the highly halophilic environment; this mechanism ensures that the internal salt concentration is maintained at the same molarity as the external environment [10,11]. The genome of *H. volcanii* is highly polyploid, with a copy number of approximately 20 copies per cell, as well as being relatively GC rich (approx. 65%) [12,13].

In the 1980s and 1990s, ground-breaking work from the groups of W. Ford Doolittle, Moshe Mevarech and Mike Dyall-Smith developed techniques for the transformation and genetic manipulation of *H. volcanii*, enabling researchers to use this organism to study halophilic archaea [9,14,15]. Since then, a variety of genetic, molecular and biochemical tools have been developed, making *H. volcanii* one of the key model organisms within the Archaea [16].

### 1.2. Genetics, molecular biology and biochemistry tools for *H. volcanii*

— Ability to grow in complex and defined media, in both broth and agar, in a wide range of salinities;
— antibiotic selection including novobiocin resistance and mevinolin resistance [15,17];
— auxotrophic selection including selectable markers for uracil, leucine, tryptophan and thymidine biosynthesis [18–20];
— efficient markerless gene deletion methods based on selection for uracil biosynthesis and counter-selection of resistance to 5-fluoroorotic acid [18,19];
— reporter genes including ß-galactosidase [21], GFP and related fluorescent proteins [22,23], and luciferase [24];
— shuttle vectors based on different *H. volcanii* replication origins [17,19,25,26];
— inducible gene expression based on a tryptophan-inducible promoter [27], and constitutive gene expression using a strong synthetic promoter [28,29];
— random genome insertion mutagenesis library [30];
— utilization of own CRISPR system as a method of gene interference (CRISPRi) [31,32];
— natural gene transfer system (cell mating), which can be used for combining mutations [14,33,34];
— genome sequence with manually curated annotation [35];
— protein overexpression and purification, and other biotechnology applications [29,36];
— proteomic methods using metabolic labelling (SILAC) along with pulse-chase lipid analysis [16,37];
— mapping of post-translational modifications [38];
— pioneer species in the Archaeal Proteome Project (ArcPP) [39].

The ease with which *H. volcanii* can be cultured in broth and on solid media, and the extensive range of genetic, molecular and biochemical tools that have been developed, have made this organism ideal to compare and contrast fundamental cellular processes with other halophiles, other archaea and other domains of life. Here, we focus on DNA replication and repair pathways in archaea, and in particular in *H. volcanii.* The knowledge gained on mechanisms of DNA replication and repair in *H. volcanii* has highlighted both similarities and differences to bacteria and eukaryotes, and has contributed to an appreciation of the diversity (and grandeur) in this view of life.

## 2. DNA replication

**Figure 1.**
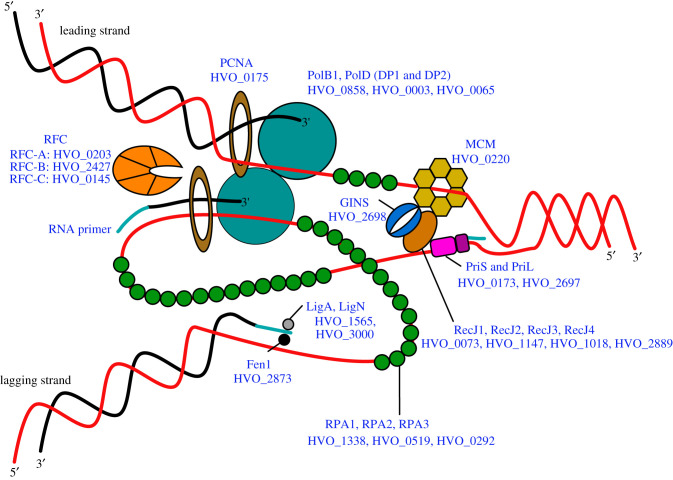
Structural components of the replisome. The CMG replicative helicase complex (RecJ:MCM:GINS in *H. volcanii*) unwinds DNA to expose single-stranded DNA (ssDNA). It remains unknown which of the four RecJ proteins in *H. volcanii* forms part of the CMG complex. The ssDNA is protected from damage by binding protein RPA and is used as a template for the synthesis of RNA primers by the primase activities of PriS and PriL. Replicative DNA polymerases (PolB1 and PolD) extend the RNA primer to initiate DNA replication. Clamp loader RFC removes primases from the replication fork and the open DNA structure is held in place by the sliding clamp PCNA. *H. volcanii* gene loci (HVO_#) for each component of the replisome are indicated.

DNA replication is a fundamental cellular process and can be divided into three stages: initiation, elongation and termination. The initiation of DNA replication occurs at specific chromosomal sites termed origins and relies on the binding of initiator proteins at these sites [40]. Origins contain AT-rich sequences named duplex unwinding elements (DUEs), where weaker hydrogen bonding facilitates DNA strand opening. Binding of initiator proteins triggers the recruitment of a helicase that, when active, further unwinds the DNA double helix, exposing single-stranded DNA (ssDNA; outlined in figure 1 and table 1). The ssDNA is protected by single-stranded DNA-binding proteins (SSBs) that have an additional role in the downstream recruitment of replication factors, including primases and DNA polymerases. The formation of a replisome complex initiates bidirectional DNA synthesis in opposing directions away from the origin. During elongation, primases generate short RNA primers from which DNA polymerases prime synthesis of the leading strand continuously in a 5′–3′ direction, while replication of the lagging strand occurs discontinuously via the formation of Okazaki fragments. Additional components of the replisome include clamp loader proteins, which act to load sliding clamp proteins that act both as a molecular toolbelt and processivity factor for DNA polymerases. Termination of DNA replication occurs when replication forks meet and resolve, allowing for correct chromosome segregation upon completion of DNA synthesis.

**Table 1.** DNA replication and repair enzymes and gene loci in *H. volcanii*.

process	function	enzyme	HVO_ gene locus	notes
replication initiation	origin binding	Orc1	0001	*oriC1*
		Orc2	0634	*oriC3*
		Orc3	A0001	*ori-pHV4*
		Orc5	1725	*oriC2*
		Orc6	B0001	*ori-pHV3*
		Orc10	C0001	*ori-pHV1*
replisome formation	CMG replicative helicase complex	MCM	0220	
		GINS	2698	
		RecJ1	0073	alternative GAN proteins, Cdc45 orthologue not yet determined
		RecJ2	1147	
		RecJ3	1018	
		RecJ4	2889	
	primer generation (primase)	DnaG	2321	‘bacterial’ primase, unlikely to act in replication
		PriS	2697	‘eukaryotic’ primase
		PriL	0173	
	clamp loader	RFC-A	0203	
		RFC-B	2427	
		RFC-C	0145	
	clamp protein	PCNA	0175	
	single-stranded DNA-binding protein	RPA1	1338	only RPA2 essential, RPA1/3 unlikely to play major role in replication
		RPA2	0519	
		RPA3	0292	
	DNA ligase	LigA	1565	alternative and redundant ligases
		LigN	3000	
DNA synthesis	replicative DNA polymerase	PolB1	0858	
		PolD1 (DP1)	0003	small exonuclease subunit of PolD
		PolD2 (DP2)	0065	large subunit of PolD
termination of DNA replication	dimer and superhelical torsion resolution	XerC-like	1422	involvement in termination of replication yet to be shown
			2259	
			2273	
			2290	
		TopoIA	0681	‘bacterial’ topoisomerase
		TopoVI-A	1570	‘archaeal’ topoisomerases
		TopoVI-B	1571	
		GyrA	1572	‘bacterial’ topoisomerases
		GyrB	1573	
	removal of RNA primers from replicated DNA/DNA:RNA hybrids	RNaseH-A	2438	Type I RNase H
		RNaseH-B	1978	Type II RNase H
		RNaseH-C	A0463	Type I RNase H
		RNaseH-D	A0277	
		RNaseH-E	0732	Type I RNase H
	flap endonuclease	Fen1	2873	also acts in various repair pathways
direct DNA repair	photolyase	Phr1	2911	
		Phr2	2843	
		Phr3	1234	as yet uncharacterized
	type IV restriction enzyme	Mrr	0682	
	methyltransferase		A0006	targets cytosine at C^m4^TAG motifs
		Zim	0794	
			A0237	
		rmeRMS	2269–2271	targets adenine at GCA^m6^BN_6_VTGC motifs
base excision repair	DNA glycosylase	Udg1	0231	uracil DNA glycosylase
		Udg2	2792	
		Udg3	0444	
		Udg4	1038	
		OGG	1681	DNA N-glycosylase
		AlkA	2814	DNA-3-methyladenine glycosylase
		MutY1	2896	A/G-specific adenine glycosylase
		MutY2	2834	
	AP endonuclease	Apn1	0573	endonuclease IV
		NthA	0848	endonuclease III
		NthB	0878	
		EndIV	2708	
		EndV	0726	
		EndVb	0443	endonuclease V homologue
nucleotide excision repair	damaged DNA recognition	UvrA	0393	
	helicase	UvrB	0029	
	endonuclease	UvrC	3006	
	helicase	UvrD	0415	redundant function with other helicases
mismatch repair	predicted ATPase	MutLa	1939	active in mismatch repair
		MutLb	0551	
	mismatch repair ATPase	MutS1a	1940	
		MutS1b	0552	
		MutS5a	0191	not involved in mismatch repair
		MutS5b	1354	
	branched structure endonuclease	NucS	0486	also called EndoMS
translesion synthesis	translesion polymerase	PolY	1302	
microhomology-mediated end joining (end resection)	ATPase	Rad50	0854	work together in Mre11-Rad50 complex
	exonuclease	Mre11	0853	
homologous recombination	recombinase	RadA	0104	
	recombinase mediator	RadB	2383	
	strand displacement	Hel308	0014	
		Hef	3010	
	Holliday junction resolvase	Hjc	0170	alternative and redundant resolvases
		Hef	3010	

### 2.1. Initiation of DNA replication

Bacteria generally have a single circular chromosome with a single origin of replication, *oriC*. Initiation of replication begins when initiator protein DnaA binds *oriC* at sequence-specific sites called DnaA boxes. The cooperative binding of DnaA forces open the duplex at the DUE, forming a ssDNA bubble [41], while bacterial SSB binds to the exposed ssDNA. The helix opening at *oriC* allows access to the helicase loader DnaC, which acts as a chaperone to recruit replicative helicase DnaB onto the lagging strand. Activation of the helicase is dictated by DnaC; when DnaC is bound by ATP, DnaB is inactive, but when DnaC is bound by ADP DnaB helicase is activated [42,43]. Active DnaB unwinds double-stranded DNA (dsDNA), increasing the size of the replication bubble and allowing downstream recruitment of the remainder of the replication components including primase, DNA polymerase and clamp protein ß. Only a single hexamer of DnaB is loaded per replication fork [44].

DNA replication initiation in eukaryotes is inherently more complex than in bacteria; multiple origins are present along the length of multiple linear chromosomes, with initiation being triggered by the binding of a multimer of initiation proteins known as the origin recognition complex (ORC). The ORC complex consists of six origin recognition proteins (termed Orc1-Orc6) [45]; Orc1–5 proteins contain a winged-helix (WH) domain that facilitates their binding at the origin [46]. Prior to S-phase, the ORC complex, together with the regulator cell division cycle 6 protein (Cdc6) and the licensing factor Cdc10-dependent transcript 1 protein (Cdt1), load the replicative helicase mini-chromosome maintenance (MCM2–7; consisting of 6 paralogous proteins) to form the pre-replicative complex (pre-RC) [47,48]. The ATPase AAA+ domains of Orc1–5 initiator proteins interact with the C-terminal WH domain of MCM in an ATP-dependent reaction. Any exposed ssDNA is coated with eukaryotic SSB protein, named replication protein A (RPA), for protection. Upon recruitment to the pre-RC, MCM helicase is inactive; activation must occur for elongation to begin. Activation of the replicative complex occurs in S phase, whereupon ORC, Cdc6 and Cdt1 are no longer required and will dissociate. MCM helicase is loaded onto the leading strand and, unlike the situation in bacteria, multiple MCM molecules can associate with a single replication fork [49,50].

Archaea have circular chromosomes and can use single or multiple origins to initiate DNA replication [51]. Despite the similarity in genome organization between archaea and bacteria, DNA replication mechanisms used by archaea differ widely from those used in bacteria; archaeal cells possess eukaryotic-like replication mechanisms employing multiple origins and Orc1/Cdc6-like proteins (referred to onwards as Orc).

Similar to bacterial and eukaryotic origins, archaeal replication origins are AT-rich regions (DUE), but in archaea are surrounded by origin recognition box (ORB) sequences, of which pairs are often inverted around the DUE [52]. ORBs direct the binding of Orc proteins onto DNA, with one Orc monomer binding a single ORB sequence with a defined polarity. ORBs are found on the minor groove of DNA and contain a signature string of guanine nucleotides known as the G-string [53]. Strand opening enables stable binding of the N-terminal AAA+ domain of the Orc protein(s) to the G-string, while the C-terminal WH DNA-binding domain of the Orc protein determines the binding affinity to the origin through binding to the ORB more generally [54,55].

Orc binding at the origin does not cause further melting of the dsDNA; instead, it facilitates the recruitment of MCM helicase (a single polypeptide in archaea, homologous to eukaryotic MCM2–7) to mark the origin for replisome loading [51,52]. Only the ATP-bound Orc is capable of MCM recruitment, while ADP-bound Orc is unable to sustain interactions with MCM [52,56]. Archaeal MCM forms a homohexameric ring, which is loaded onto the leading strand of DNA as a double hexamer, the pair of inverted Orc proteins surrounding the DUE each load a single hexamer of MCM.

An initial study on archaeal DNA replication mechanisms mapped the single replication origin of *Pyrococcus abyssi* using nucleotide skew analysis [57]. Since then, more advanced techniques, including two-dimensional gels, whole-genome microarrays and marker frequency analysis (MFA), have enabled the identification and mapping of origins in over 20 archaeal species (reviewed in [58]). It is now clear that the number of origins and Orc proteins varies considerably throughout the archaea [40], but one of the striking consistencies is that the gene encoding the Orc protein is (nearly) always found directly adjacent to its cognate origin. The association of origin and Orc in close proximity allows independent control of each origin and reduces competition between origins and initiators [56].

The origins of replication for *H. volcanii* were first mapped in 2007 [59]. The genomic architecture of *H. volcanii* consists of one main circular chromosome (2.8 Mb) and three circular mini-chromosomes; pHV4 (636 kb), pHV3 (438 kb) and pHV1 (85 kb) [35]; the 6 kb plasmid pHV2 has been cured from the laboratory strain and its derivatives [15]. The main chromosome features three active origins, each associated with their own corresponding Orc initiator protein (*oriC1* is associated with Orc1, *oriC2* with Orc5 and *oriC3* with Orc2) [35,59]. As with the main chromosome, each mini-chromosome of *H. volcanii* has its own origin and corresponding Orc protein [40]. Origins vary in activity; *oriC1* the most active origin in *H. volcanii* [60] and deletion of *oriC1* and its corresponding Orc protein Orc1 results in a reduced ploidy, suggesting an additional regulatory role for this origin [61,62]. Subsequent studies have confirmed these initial findings and the replication profile of the main chromosome of *H. volcanii* is now mapped in detail [60,63].

The laboratory strain of *H. volcanii* contains a fourth replication origin on the main chromosome, *ori-pHV4* (and its corresponding Orc protein, Orc3). A fusion event between insertion sequence (IS) elements on pHV4 and the main chromosome led to the stable integration of pHV4 and thus a newly acquired main chromosomal origin [60]. Additional stable genomic rearrangements in *H. volcanii* have been observed, where a recombination event between two near-identical *sod1* and *sod2* genes led to the creation of a stable mini-chromosome [64].

Alongside the three origin-associated Orc proteins in *H. volcanii*, there are 12 additional Orc proteins whose genes are not linked to origins. Their function is currently unknown, but they are most likely to be dormant Orc proteins that have been orphaned due to the integration of foreign genetic elements. For example, the genes encoding Orc11 and Orc14 are both located within a 50 kb prophage region [35]. Such integration events, coupled with the fluctuating genome configurations of archaeal species, hints at evolutionary mechanisms that have facilitated a multi-origin replication system, including horizontal gene transfer (HGT) and gene duplication events [61,65]. For example, the replicon takeover hypothesis postulates that the host chromosome becomes dependent on extra-chromosomal elements for its propagation [66]. The apparent fluidity of the *H. volcanii* genome architecture provides a tool for study of how the loss or gain of an origin and/or Orc can lead to the multi-origin chromosomes seen in several archaeal species.

### 2.2. Elongation

For DNA replication to proceed away from the origin, a full replisome must be established. All domains of life share basic mechanisms of DNA synthesis, but differ primarily in the proteins used [67,68]. In eukaryotes, the pre-replication complex (pre-RC) must be activated prior to elongation; this activation provides a further level of regulation above that of bacteria.

Formation of a replisome in bacteria is relatively simple; following activation of DnaB helicase, the remaining components are recruited in a stepwise manner. Primase DnaG, a DNA-dependent RNA polymerase, acts to synthesize short (approx. 8–12 nucleotide) primers used by DNA polymerase to prime synthesis [69]. Pol-III is the main replicative polymerase in bacteria, while Pol-I is involved in Okazaki fragment maturation (discussed in more detail later). Bacterial clamp protein ß ensures Pol-III remains associated with the template and increases the processivity of the polymerase during synthesis.

In eukaryotes, the activation of MCM2–7, and therefore activation of the pre-RC, is dependent upon two events: phosphorylation by kinases cyclin-dependent kinase (CDK) and Dbf4-dependent kinase (DDK) [70], and the formation of the CMG replicative helicase complex (consisting of Cdc45, MCM and GINS proteins). Assembly of the CMG complex in eukaryotes causes switching of MCM binding from dsDNA to ssDNA, activating the helicase for helix unwinding [71,72]. At this point, further replication components can be loaded, such as replicative DNA polymerases (Pol-α, Pol-δ and Pol-ε), primases (PriS/L) and clamp protein proliferating cell nuclear antigen (PCNA). Primase acts in complex with Pol-α (as complex PrimPol) to synthesize an approximately 30 bp RNA-DNA primer for extension by Pol-ε to synthesize the leading strand, while Pol-δ performs synthesis of the lagging strand.

Elongation in archaea is akin to the more complex system of eukaryotes: MCM associates with Cdc45-like and GINS-like proteins to form an archaeal CMG complex, following which the remainder of replication components are loaded and replication proceeds bidirectionally away from the origin. Archaeal replication components will be discussed in further detail below.

#### 2.2.1. The CMG replicative helicase complex

##### 2.2.1.1. Mini-chromosome maintenance helicase

Despite the ubiquitous function of MCM helicases, there is significant genetic and structural diversity within this family of proteins. Most archaeal species encode a single MCM homologue thought to act as the replicative helicase [73]. Where species encode more than one *mcm* homologue, such as *Thermococcus kodakarensis* and *Methanococcus maripaludis*, which possess three and four MCM homologues, respectively, only one will be essential for viability [74–76]. The essential MCM protein in species with multiple paralogues shares structural and sequence similarity with the single MCM proteins in other species [75,76].

As a hexamer, archaeal MCM possesses 3′–5′ helicase activity that opens the DNA duplex while translocating along the leading strand. Archaeal MCM proteins are members of the AAA+ ATPase superfamily and are made up of a non-catalytic N-terminal domain, a central catalytic AAA+ domain and a C-terminal winged-helix-turn-helix (wHTH) domain [77]. However, structural variations of archaeal MCM have been found with some homologues lacking an N-terminal domain or helicase activity, while others consist only of a partial C-terminal domain [73,78]. The N-terminal portion is important for hexamer formation, enzyme regulation and DNA binding [73]. The catalytic region contains residues associated with other AAA+ ATPases, with Walker A and Walker B motifs being required for ATP binding and hydrolysis, respectively. The presence of an arginine finger motif within the catalytic domain is characteristic of MCM as a protein of this superfamily, with the string of positively charged residues instigating a strong interaction with negatively charged DNA [77]. At the interface of N-terminal and catalytic domains is the allosteric control loop (ACL); the ACL consists of a β7–β8 β-hairpin loop and acts to regulate interactions between the N- and C-terminus of MCM [79,80]. The C-terminal wHTH domain is implicated in the regulation of MCM but is yet to be fully characterized [73,81].

Unlike eukaryotic MCM, archaeal MCM has basal activity without the requirement for interactors Cdc45 and GINS [82–84]. Archaeal MCM proteins can form a range of structures in solution, but only hexameric MCM has been shown to possess helicase activity [85]. Double hexameric MCM has been shown to be more active than the monomeric form, suggesting double hexameric MCM acts during canonical replication [86]. The crystal structure for *Sulfolobus solfataricus* MCM has revealed a feature specific to archaea; each monomer of MCM encodes four β-hairpins, three positioned within the main channel and one externally [79]. Mutational analysis has since revealed these structures play a key role in both DNA-binding and helicase activity [78].

*H. volcanii* encodes a single essential MCM homologue (HVO_0220), which forms a homohexameric structure akin to other archaeal MCM complexes [77,87]. Structurally, *H. volcanii* MCM is made up of a zinc-binding N-terminal domain, an AAA+ catalytic core and a C-terminal wHTH domain. It uses a zinc-cofactor to break the hydrogen bonding of the DNA double helix [77]. Mutagenesis studies have shown short β7-β8 β-hairpin loop deletions and large β9-β10 β-hairpin loop deletions within the N-terminal domain are intolerable to the cells; it is speculated that these loops are crucial for the coordination of zinc binding [77]. Furthermore, a G187A mutation and alanine substitutions of conserved zinc-binding cysteines show these residues play a critical role in MCM function. Like other archaeal MCM homologues, it is essential for cell viability; deletion of the full-length gene is not possible (T.A. 2020, unpublished) and specific β7–β8 loop and zinc-binding domain mutants of *H. volcanii* MCM could not be generated [77].

##### 2.2.1.2. GINS complex

GINS complex (named after Japanese numbers 5-1-2-3, go-ichi-ni-san, representing subunits of the eukaryotic complex Sld5, Psf1, Psf2 and Psf3, respectively) is known to play an essential role in eukaryotic replication [88]. The four subunits of eukaryotic GINS are predicted to be paralogous [89], but can be clustered into two groupings based on protein structures; A-domains contain high amounts of α-helices, while B-domains are smaller and rich in β-strands. This structural grouping places Sld5 and Psf1 together with an AB domain organization, and Psf2 and Psf3 together with a BA domain organization [90]. Archaeal GINS complex, as with MCM, is a simplified version of the eukaryotic counterpart. Structurally, archaeal and eukaryotic GINS are comparable, but archaeal GINS is encoded by only one (*gins51*) or two (*gins51* and *gins23*) genes, depending on species [89,91,92]. Archaeal GINS51 protein shares structural similarity with Sld5 and Psf1 (AB-type), while GINS23 shares similarity with Psf2 and Psf3 (BA-type). Species can either form a dimer of dimers where GINS51 and GINS23 are encoded, or a homotetramer of GINS51 alone in the absence of GINS23.

The first archaeal GINS homologue to be identified and characterized was from *S. solfataricus.* Its GINS complex forms a tetrameric structure made up of GINS51 and GINS23 dimers. In *S. solfataricus*, these genes are found in the same operon as both MCM and primase. A clear interaction has since been demonstrated between GINS23, MCM and primase, providing evidence for a functional and eukaryotic-like CMG complex in *S. solfataricus* [84,91]*.* Eukaryotic-like GINS has also been identified and characterized in *T. kodakarensis*, where the crystal structure of GINS is directly comparable to that of human GINS complex [93]. As in *S. solfataricus, T. kodakarensis* GINS forms a dimer of dimers of GINS51 and GINS23 proteins [93].

Interactions between archaeal MCM and GINS are well documented, with GINS interaction boosting the ATPase and helicase activities of MCM. In species encoding both GINS51 and GINS23 subunits, interactions with MCM are mediated by GINS23 [91,94]. *Thermoplasma acidophilum* encodes only GINS51 subunits, forming a homotetramer, but has been shown to carry out MCM : GINS interactions in a GINS51-dependent manner [83,95]. A GINS51-MCM interaction has yet to be observed in species carrying both GINS51 and GINS23 subunits.

A single homologue of GINS has been identified in *H. volcanii*. The gene HVO_2968 encodes a GINS51-type protein and is located within an operon with primase gene *priS* (HVO_2697) [87]. Structurally, *H. volcanii* GINS is larger than the eukaryotic counterparts, due to the presence of a large sequence insertion between the conserved A and B domains. Such an insertion is seen in numerous halophilic species; however, the function of such an insertion remains unknown and warrants further study [92]. As yet, interactions between *H. volcanii* GINS and other components of replication have not been described; like MCM, GINS is essential for cell viability (T.A. and Stuart MacNeill 2020, unpublished).

##### 2.2.1.3. Cdc45/recJ/GAN

While archaeal MCM and GINS homologues are easy to identify based on similarity to their eukaryotic counterparts, identification of a Cdc45-like protein in archaea has been less straightforward. Bioinformatic analysis of Cdc45 revealed it is the eukaryotic orthologue of bacterial and archaeal RecJ phosphodiesterase/nuclease family proteins [96,97]. Specifically, the N-terminus of eukaryotic Cdc45 shows similarity to the DHH domain associated with RecJ family nucleases [98]. However, unlike well-characterized RecJ proteins, Cdc45 is known to lack catalytic activity; this can be explained by the loss of key catalytic residues within the DHH domain [96,99]. Instead, eukaryotic Cdc45 plays an essential structural role within the CMG complex. Akin to RecJ nucleases, eukaryotic Cdc45 has maintained the ability to bind ssDNA [100], which may account for its role at the replication fork.

Bacterial RecJ is relatively well characterized; it has been implicated in a number of DNA repair pathways, including mismatch repair (MMR), homologous recombination (HR) and base excision repair (BER), along with a role in the restart of stalled replication forks [101–104]. Bacterial RecJ is composed of an N-terminally located catalytic core, made up of DHH and DHHA1 domains, and a C-terminal oligonucleotide-binding (OB)-fold domain responsible for binding ssDNA. DHHA1 is a subfamily of DHH superfamily proteins, found in both bacteria and archaea but absent from eukaryotes; this subfamily domain is involved in substrate specificity [105,106]. The C-terminal positioning of the OB-fold is specific to bacterial RecJ and is not found within Cdc45 or archaeal RecJ proteins.

The DHH superfamily has undergone an expansion event within the archaea (specifically within the phylum Euryarchaea), where multiple species now encode several RecJ-like proteins. To date, every archaeal species studied encodes at least one RecJ protein [107] and while some homologues retain previous identities, others evolved quickly and developed novel roles [96]. RecJ proteins in archaea have now been implicated in DNA repair and replication, some have lost all activity, while the roles of many remain unknown [96].

RecJ proteins were first implicated in archaeal DNA replication following the identification of *in vivo* interactors with a range of replication components in *T. kodakarensis* [108]. Two RecJ-like proteins were identified in *T. kodakarensis*, termed GAN (GINS-associated nuclease) and HAN (Hef-associated nuclease) [109,110]. GAN was primarily identified as an *in vivo* interactor with GINS, interacting specifically with the GINS51 subunit, while HAN interacted with stalled fork repair protein Hef, favouring GAN as the Cdc45-like protein in *T. kodakarensis* [110–112]. Since its discovery in *T. kodakarensis*, GAN homologues have been identified in various archaeal species, reflecting the complexity and diversity of archaeal DNA replication factors [96]. RecJ-like/GAN proteins are thought to be the Cdc45-like protein within the CMG complex of archaea (also called GMG for GAN : MCM : GINS). For all characterized archaeal CMG complex interactions mapped to date, GINS is essential to bridge the interaction between MCM and Cdc45 [82–84].

The GAN : GINS complex acts to boost the helicase activity of MCM, akin to the role of Cdc45 in eukaryotic CMG complexes [82]. The crystal structure of GAN has revealed similarities to eukaryotic Cdc45; the DHH domain contains a CID (CMG-interacting domain), as in Cdc45 [109,113]. However, unlike its eukaryotic counterpart, GAN remains catalytically active as a processive 5′–3′ ssDNA exonuclease [112]. GAN has been shown to form a complex with GINS and MCM *in vitro*, and the interaction with GINS51 stimulates the exonuclease activity of GAN [82]. By contrast, the alternative RecJ protein HAN does not interact with GINS [110], suggesting an alternative role for this RecJ homologue. It has recently been shown that the *T. kodakarensis* replicative DNA polymerase subunit PolD2 interacts with GAN via the GINS complex, placing GAN at the heart of the replication complex as in eukaryotes [108,109,112,114].

Similar to *T. kodakarensis*, *Pyrococcus furiosus* encodes two RecJ-like proteins, one sharing sequence and structural similarity with GAN (PF2055; here called *Pfu*RecJ) and the other sharing characteristics with HAN (PF0399). The DHH domain of *Pfu*RecJ is intact, facilitating 5′–3′ DNA exonuclease activity, 3′–5′ RNA exonuclease activity, and interactions with GINS51, implicating *Pfu*RecJ in DNA replication as a member of the CMG complex. Interaction of *Pfu*RecJ and the SSB protein RPA results in 3′–5′ exonuclease activity on both ssRNA and dsRNA/DNA hybrids; such an activity could be used at the replication fork to deal with Okazaki fragments [115,116]. The crystal structure of *Pfu*RecJ has been solved and is comparable to that of *T. kodakarensis* GAN [116]; *Pfu*RecJ is therefore is a strong candidate for the Cdc45-like protein in *P. furiosus*.

However, this pattern is not observed in all Euryarchaea. Two RecJ homologues, *Ta*RecJ1 and *Ta*RecJ2, have been identified in *Thermoplasma acidophilum,* both bearing resemblance to *T. kodakarensis* GAN [117]. *Ta*RecJ1 possesses 5′–3′ ssDNA-specific exonuclease activity, while *Ta*RecJ2 possesses 3′–5′ exonuclease activity specific for RNA. Interactions between *Ta*RecJ2 and GINS51 occur in a stable fashion and it has been shown that *Ta*RecJ2, not *Ta*RecJ1, in combination with GINS, stimulates the helicase activity of *T. acidophilum* MCM [117]. A CMG-like complex comprising *Ta*RecJ2 : MCM : GINS was recapitulated *in vitro* and also observed *in vivo*, making *Ta*RecJ2 the true ‘GAN’ of this species [117]. The 5′-3′ directionality of *Ta*RecJ1 is akin to that of bacterial RecJ, and it is possible that *Ta*RecJ1 has gained a role in DNA repair akin to bacterial RecJ; this is yet to be confirmed.

Unlike the full-length RecJ proteins acting as Cdc45 in the aforementioned examples, the crenarchaeon *S. solfataricus* instead uses a truncated form of RecJ [91]. Primarily identified through its interaction with GINS51, RecJdbh (RecJ DNA-binding homologue) or *‘*Cdc45’ shares only the DNA-binding portion of bacterial RecJ. These ‘inactive’ RecJ proteins have been shown to form CMG-like complexes and boost the helicase activity of MCM, making them *bona fide* Cdc45-like proteins [84,91]. RecJdbh shares very little homology to characterized GAN proteins, carrying a degenerate DHH domain and lacking any exonucleolytic activity. This suggests that there is more than one type of RecJ protein able to act in the manner of Cdc45 in archaea and warrants further study.

The identity of the Cdc45 homologue in *H. volcanii* is also an open question. Preliminary work has identified four RecJ homologues: RecJ1 (HVO_0073), RecJ2 (HVO_1147), RecJ3 (HVO_1018) and RecJ4 (HVO_2889) [107]. Based on similarity to Cdc45, GAN and RecJ proteins in general, it has been proposed that one (or more) of these RecJ homologues function as the Cdc45 homologue in *H. volcanii* [71]. Analysis of catalytic residues within RecJ1 and RecJ3 suggest they have retained nuclease activity, but *in vitro* and *in vivo* studies are needed to determine whether these nucleases are active, and whether they act in DNA repair or replication. By contrast, RecJ2 and RecJ4 are both predicted to have lost key catalytic residues and therefore nuclear activity. Comparison of *H. volcanii* RecJ proteins by arCOG grouping (archaeal clusters of orthologous genes) suggests that RecJ1 is the best candidate for GAN, while RecJ3 and RecJ4 are HAN candidates; RecJ2 has diverged away from the other *H. volcanii* RecJ proteins and its function remains unknown. RecJ1, RecJ3 and RecJ4 are all non-essential (even in combination) but the cellular requirement for RecJ2 is an open question (T.A. and Stuart MacNeill 2020, unpublished). Further work is needed to decipher which RecJ(s) play the role of Cdc45 and whether there is any redundancy between the four RecJ proteins in *H. volcanii*.

#### 2.2.2. Other replisome components

##### 2.2.2.1. Primases

Bacteria use a single subunit primase protein, DnaG, while eukaryotes encode heterodimers consisting of catalytic (PriS/p48) and regulatory (PriL/p58) subunits that work in tandem to synthesize short primers [69,118].

Archaea encode both bacteria-like and eukaryotic-like primases, depending on the species. The eukaryotic-like replicative primase found in archaea is a two-subunit complex consisting of a small catalytic subunit (PriS/p41) and a large regulatory subunit (PriL/p46); fusion events of PriS and PriL have been seen within nanoarchaeal genomes [61,68]. Unlike bacterial and eukaryotic primases that are only capable of synthesizing ribonucleotides, archaeal primases have been shown capable of synthesizing both RNA and DNA [119]. DNA synthesis can reach lengths of several kilobases, meaning archaeal primases in some cases can be defined as non-canonical DNA polymerases [119,120]. PriS/L-like primases in archaea have also been implicated in functions outside of replication, including gap-filling and strand displacement activities [121]. Bacterial-like DnaG proteins in archaea do not appear to act in DNA replication. For example in *S. solfataricus*, DnaG has been strongly implicated in RNA degradation and has only limited primer synthesis activity [122,123]; instead, *S. solfataricus* uses PriS/L to carry out primer synthesis during DNA replication [123,124].

*H. volcanii* also encodes homologues of both bacterial and eukaryotic primases. Bacterial DnaG primase (HVO_2321) can be deleted from *H. volcanii* without any effect on cell viability [121], suggesting that this protein has no role in DNA replication. As in *S. solfataricus,* DnaG may have gained an alternative role in RNA degradation but this requires further testing. By contrast, eukaryotic-like PriS and PriL genes (HVO_2697 and HVO_0173 respectively) are essential for cell viability [121], most likely priming DNA replication at the replisome. Significant work on the activities of PriS/L is still needed, including the length of RNA/DNA primers synthesized, polymerase specificity and any additional roles in the cell.

##### 2.2.2.2. Clamp loader replication factor C

Sliding clamp proteins are required to boost the otherwise low processivity of replicative DNA polymerases. Sliding clamps are stable ring proteins and thus cannot self-assemble onto DNA; instead, they are assembled onto DNA by a clamp loader protein [125]. Clamp loader proteins facilitate the opening and loading of the clamp protein (ß-clamp protein in bacteria, PCNA in eukaryotes and archaea) onto a primer-template junction in an ATP-dependent manner [126]. Bacteria use clamp loader γ-complex while eukaryotes and archaea rely on replication factor C (RFC). The ability of clamp loader proteins to distinguish ssDNA : dsDNA junctions allows loading of clamp proteins specifically at primer-template junctions [126]. The primase, at the time of clamp recruitment, remains associated with the primer. Both primases and clamp loaders interact with SSB and this facilitates the handoff from primase to clamp loader protein binding the primer-template junction [127]. Due to the discontinuous priming of the lagging strand, clamp proteins are continuously recruited, meaning there is a constant requirement for clamp loaders during processive replication [128].

Eukaryotic RFC is pentameric and is composed of one large subunit (Rfc1) and four small subunits (Rfc2–5). Most archaea encode two homologues of RFC: one corresponding to the small eukaryotic RFC subunit (RFCS) and the other corresponding to the large subunit (RFCL) [44,125]. Akin to eukaryotic RFC, archaeal RFC forms a pentamer consisting of four RFCS subunits and one RFCL subunit [129]. Stimulation of PCNA by RFC has been characterized in *Pyrococcus horikoshii*, whereby RFC enables PCNA to recruit and activate both replicative DNA polymerases [130,131].

*H. volcanii* possesses three homologues of RFC (RFC-A, HVO_0203; RFC-B, HVO_2427; RFC-C, HVO_0145), all of which are essential for growth [132]. All three homologues possess AAA+ domains that enable ATP-dependent DNA binding. The larger of the three, RFC-B, carries an additional C-terminal PIP box that is absent from the smaller RFC subunits. A PIP box (PCNA-interacting protein peptide box, discussed in more detail later) facilitates interaction with PCNA, suggesting that RFC-B acts to stimulate PCNA for polymerase recruitment. Further work is required to decipher the roles of the RFC subunits in *H. volcanii*.

##### 2.2.2.3. Proliferating cell nuclear antigen

The clamp protein proliferating cell nuclear antigen (PCNA) acts as a platform for the recruitment of DNA polymerases and other replicative proteins in eukaryotes and archaea. The protein binds dsDNA in a sequence-independent manner where it can move bidirectionally. PCNA acts as a clamp at the replication fork to tether replication factors onto DNA via the opening and closing of its ring structure around dsDNA (aided by clamp loader protein). Bacteria have a differing clamp protein, named *β*, which forms a homodimer, while both eukaryotes and archaea use trimeric protein PCNA [125].

Regarding clamp proteins in archaea, there appears to be a division along phylogenetic lines: in most euryarchaea, there is a single PCNA homologue that forms a homotrimer. Only one euryarchaeal species, *T. kodakarensis,* carries two PCNA homologues; however, one is believed to have been acquired relatively recently by lateral gene transfer (LGT) [133]. On the other hand, crenarchaea commonly encode multiple PCNA homologues and have been shown to form both homo- and hetero-trimeric structures [134].

*H. volcanii*, as a euryarchaeon, encodes a single homologue of PCNA (HVO_0175). PCNA is essential for viability in *H. volcanii* and forms a homotrimer in solution, with monomers interacting in a head-to-tail manner [135–137]. *H. volcanii* PCNA has been predicted to interact with numerous replication components, including replicative DNA polymerases PolB1 and PolD, clamp loader protein RFC-B, endonuclease Fen1 and ribonuclease RNase H2 [137]. All of these proteins contain a PIP box, a defined region of the protein made up of bulky aromatic groups containing conserved residues QxxLxxFx (where x represents any amino acid) [137]. Interactions of PCNA with proteins via PIP boxes is well-characterized throughout archaeal and eukaryotic species [68,138] and underlines a key role for PCNA in DNA replication. Alongside its role in replication, PCNA has also been linked to proteins associated with DNA repair via the identification of PIP boxes; these links are discussed in detail later. The ability of PCNA to interact with multiple proteins simultaneously has given rise to the ‘molecular toolbelt’ model, where PCNA acts to bring together replication and repair proteins at the site they will be required.

Structural studies of PCNA in *H. volcanii* have advanced our knowledge of protein adaptation to high intracellular salt concentrations [135,136]. Bacterial and eukaryotic PCNA homologues feature positively charged residues (commonly lysine and arginine) in the two α-helices that make up the inner channel of the ring structure. This facilitates strong interactions between PCNA and negatively charged DNA. Due to the high internal salt concentration of *H. volcanii* cells, proteins have adapted by increasing their surface acidity (specifically by increasing the percentage of aspartate and alanine residues), along with increasing the number of bound cations and intermolecular ion pairs. The crystal structure of *H. volcanii* PCNA shows a notable increase in surface acidic residues to alter the overall electrostatic charge distribution of the protein. This enables the protein to function with only two basic residues per monomer in the channel. *H. volcanii* PCNA also has increased cation binding to potentially facilitate a reduction in positively charged atoms at the pore region with three Na^+^ ions over two sites in each monomer. These adaptation mechanisms enable PCNA be stable at a wide range of salt concentrations while still facilitating the critical interaction of PCNA with DNA.

Interestingly, the deletion of proteasome-activating nucleotidase A (PanA; HVO_0850) increases the half-life of PCNA, demonstrating that *H. volcanii* PCNA is a target of proteasomal degradation [139,140]. This study, using pulse-chase labelling, immobilized metal affinity chromatography (IMAC) and immunoprecipitation, is one of the first to demonstrate any post-translational regulatory mechanisms during DNA replication in *H. volcanii*. It is suggested that post-translational phosphorylation events may also target *H. volcanii* PCNA as these same techniques purify phosphopeptides. Significant work needs to be carried to understand the intricacy of post-translational events occurring in *H. volcanii*.

##### 2.2.2.4. DNA polymerases

Replicative DNA polymerases (DNAPs) function in a 5′ to 3′ manner, extending RNA primers to replicate the genome. Due to their directionality, synthesis of the leading strand is a continuous process, requiring only a single priming event, while the lagging strand must be synthesized discontinuously as Okazaki fragments.

Based on amino acid sequence, DNAPs were assigned to six main families: A, B, C, D and Y [141]. More recently, reverse transcriptase (RT) enzymes have also been defined as DNA polymerases of a separate novel family [142]. Replicative DNAPs used in each of the three domains differ, spreading across families A, B, C and D [143]. The identity and role of bacterial and eukaryotic replicative polymerases are relatively well defined. Although the families of DNAPs used by archaea have been identified, the definition of which replicative polymerase acts on which strand still remains a matter of contention.

Genome replication in bacteria is reliant on Pol-III (Family C polymerase) [141]. Two copies of Pol-III replicate both the leading and lagging strands simultaneously. The Pol-III core is tightly associated with the replisome via interactions with both clamp loader *γ* and clamp protein ß. Alongside the catalytic subunit, Pol-III also encodes subunits possessing 3′–5′ exonuclease proofreading activity. Gram-negative bacteria with a low GC content use two distinct copies of Pol-III, named PolC and DnaE, for leading and lagging strand synthesis, respectively [144,145]. Bacteria also encode family A polymerases, such as Pol-I. These function primarily in the processing and maturation of Okazaki fragments and removal of RNA primers [146].

Eukaryotes can encode up to 15 family B DNA polymerases. The main eukaryotic replicative DNAPs fall within this family, named Pol-α, Pol-ε and Pol-δ. These are all multi-subunit enzymes containing a catalytic core identifiable as a family B polymerase, alongside various accessory domains depending on the polymerase [147]. PrimPol generates a short RNA primer, which is then extended for approximately 40 nucleotides by low-fidelity Pol-α [68,148]. The bulk of synthesis is completed by high-fidelity replicative polymerases Pol-ε and Pol-δ [67,149,150].

Interestingly, there is a phylogenetic divide in the distribution of DNAP families in the archaea. While all archaea possess family B polymerases, archaeal-specific family D DNAPs are absent from crenarchaeal species. Work is beginning to elucidate the roles and functions of these polymerases but the question of which polymerase(s) acts at the leading and/or lagging strand remains under dispute.

Archaeal family B polymerases share homology with the catalytic subunit of family B replicative polymerases in eukaryotes [151,152]. Archaeal family B polymerases have been isolated, with some now being routinely used for PCR applications [153]. Three groups of archaeal PolB polymerases exist, historically termed PolB1, PolB2 and PolB3 [154]. PolB1 and PolB3 are active polymerases, while PolB2 proteins generally carry disrupted catalytic and exonuclease domains which can result in either an active or inactive PolB2 protein [154–156]. A single species can encode single or multiple copies of PolB, with all archaea encoding at least one PolB polymerase. It is usually present as a single protein, with one polypeptide encoding both the catalytic and proofreading activities; the exception is *Methanothermobacter thermautotrophicus*, where PolB is encoded by two polypeptides [154,157]. The distribution of specific PolB proteins changes throughout the archaeal domain; PolB1 is missing in Euryarchaeota and PolB3 is missing in Thaumarchaeota, while members of the PolB2 group are scattered across archaea [154]. Several groups of archaea carry multiple inteins within PolB3 genes, sometimes up to three per gene [158]. Inteins are selfish genetic elements that insert themselves into a coding sequence and self-splice once translated; they typically encode an endonuclease that propagates further intein insertions [159,160]. Across species carrying PolB3 proteins, intein insertion sites are generally conserved; however, some are lineage-specific [87,158].

Since Crenarchaeota species possess only family B polymerases, it is hypothesized that PolB alone must be capable of both leading and lagging strand synthesis [154,161]. However, crenarchaea typically possess multiple family B polymerases; it is possible that the multiple PolB polymerases within a strain have gained specialized functions and act on different strands. This is known to be the case for *S. solfataricus*: PolB1 (Dpo1) has been implicated in leading strand synthesis, while PolB3 (Dpo3) has been for lagging strand synthesis.

Archaeal family B DNAPs generally feature a polymerase core (made up of three domains; palm, fingers and thumb), an N-terminal 3′–5′ exonuclease domain and an uracil-recognition domain specific to archaea [154,162,163]. The uracil-recognition domain provides archaea with a unique damage sensing mechanism whereby the polymerase scans ahead of the catalytic site, pausing at misincorporated uracil or hypoxanthine moieties that have escaped canonical repair by uracil-N-glycosylase [164,165]. PolB is capable of extending DNA-primed templates efficiently; however, it struggles to extend RNA primers [166]. This suggests that the inherent DNA polymerase activity of archaeal primases or, in non-crenarchaeal species, family D polymerases are used to extent RNA primers with a short DNA template, prior to handover to PolB.

Recent studies have revealed that PolB is not essential for viability in all archaea, but can be deleted in some euryarchaeal species (which also encode PolD). In *Thermococcus barophilus, T. kodakarensis* and *M. maripaludis*, it has been shown that PolB is dispensable and PolD alone is essential [167–169]. The ability to delete PolB but not PolD in these species suggests PolD has the ability to carry out both leading and lagging strand replicative DNA synthesis, while PolB may carry out DNA synthesis as part of DNA repair. Cells of *T. kodakarensis* deleted for PolB have been shown to be sensitive to gamma irradiation, consistent with the suggestion that PolB carries out DNA synthesis during homologous recombination (which would be used to repair DNA double-strand breaks) [170]. Recent *in vitro* reconstitution studies in *P. furiosus* have shown that both PolB and PolD are capable of extending RadA recombinase-primed recombination intermediates [171], but that PolB was more efficient than PolD. This activity, of extending a D-loop recombination intermediate, is consistent with the role of PolB as a DNA repair polymerase.

However, the ability to replicate in the absence of PolB is not true of all euryarchaea. In the halophile *Halobacterium* sp. NRC-1, both PolB and PolD are essential for cell viability [172] and similar findings have been made in *H. volcanii* (T.A. 2020, unpublished). It is possible that in some species PolB has gained a novel role, or that the high ploidy associated with halophiles increases demand on replication proteins in general. Further work is required to explain the differing requirements for DNA polymerases (specifically PolB) within the euryarchaea.

The family D DNAPs were initially discovered in *P. furiosus* by the Ishino laboratory, with the discovery changing the classification system for DNA polymerases [161,173,174]. Family D polymerases form heterotetramers, encoded by subunits DP1 and DP2. DP1 is a small subunit with 3′–5′ proofreading activity and is structurally similar to the exonuclease domain of eukaryotic family B polymerases, while DP2 is the catalytic subunit [175,176]. It has been shown that interaction between DP1 and DP2 is required for PolD to achieve the maximum polymerase and exonuclease activities [173,177]. DP1 is made up of a ssDNA-binding OB fold and 3′–5′ exonuclease domain (from the metallophosphatase MPP family), which functions in the proofreading and removal of erroneously incorporated nucleotides during DNA synthesis [105,178]. The catalytic fold of this calcineurin-like phosphodiesterase family subunit has recently been shown to be specific to family D polymerases [179,180].

Structurally, family D polymerases display a close resemblance to RNA polymerases (RNAPs) [181,182]. The recent publication of a crystal structure of PolD elucidated this link in further detail [175]. While DP1 shows similarity to non-catalytic subunits of eukaryotic family B polymerases, DP2 shows homology to the two-DPBB (double-psi beta barrels) ‘two-barrel’ superfamily of polymerases [175]. Members of the two-barrel superfamily include both DNA- and RNA-dependent transcriptases, along with RNA silencing RNAPs and atypical viral RNAPs [179,181–183]. PolD is the first DNAP to be placed within this superfamily, extending the repertoire of known catalytic folds able to perform DNA synthesis [151,184]. The evolutionary history of replication posits that RNA was used as a genetic material prior to DNA [143], leading to the suggestion that PolD may be the ancestral replicative DNA polymerase of the last universal common ancestor (LUCA) [184].

Early studies in *Pyrococcus* showed that archaea-specific family D polymerase PolD can efficiently extend both RNA and DNA primers [173]. More recently, it has been shown that PolD can extend RNA primers with a greater efficiency than PolB [166]. Given this information, it has been theorized that in species encoding both PolB and PolD, PolD carries out preliminary synthesis from the RNA : DNA primer before handing over to PolB for the bulk of synthesis, akin to the mechanism seen in bacteria with Pol-I and Pol-III. However, questions remain regarding this mechanism. If PolD is the lagging strand polymerase, strand displacement activity would be required to remove the primers associated with Okazaki fragments on the lagging strand. Currently, this has only been shown in *P. abyssi* [185]. PolB has been shown to have strand displacement activity, implicating it in Okazaki fragment processing [166].

The RNA extension activity of PolD, and its processivity, requires stimulation from PCNA [175,185]. Interaction of PolD and PCNA occurs at multiple sites throughout both DP1 and DP2 subunits, including a conserved PIP motif encoded at the C-terminus of DP2. Studies on *Thermococcus* species have implicated a role for PolD at the replication fork; the DP1 exonuclease subunit associates with the GINS-GAN complex via interaction with GINS51. However, this interaction inhibits the exonuclease activity of PolD. This exonuclease activity may have a function elsewhere or may be used in the removal of replication components [114]. Recently, the three-dimensional structure of the PolD-PCNA-DNA complex in *Thermococcus kodakarensis* was determined using single-particle cryo-electron microscopy (EM). It was shown that a glutamate residue at position 171 of PCNA mediates the interaction with the DP1 and DP2 subunits, locking the PolD structure into a conformation that is competent for enzymatic activity [186].

As a euryarchaeon, *H. volcanii* contains homologues of both family B and family D DNA polymerases. Two PolB homologues are found; one is an active polymerase and one is predicted to be inactive. The active PolB, PolB1 (HVO_0858), is a member of the PolB3 family of polymerases common to euryarchaea, while the predicted-inactive PolB2 (HVO_A0065) is a member of the PolB2 group associated with often inactivated polymerases [87,156]. Little work has been carried out on *H. volcanii* PolB1 thus far; a structural analysis of the role of intein present in the C-terminus of PolB1 and its associated homing endonuclease (HEN) showed the loss of the intein sequence from the *polB1* gene resulted in no growth defects, indicating this sequence has no active role in *H. volcanii* [187]. An association between a RadA recombinase-like gene and PolB2 has been observed in *Sulfolobales* [47]. The *polB2* gene of *H. volcanii* is located near a *radA*-like gene, indicating a possible link between PolB2 group polymerases and DNA repair; however, the deletion of the *polB2* gene (but not the *polB1* gene) is possible in *H. volcanii* (T.A. 2020, unpublished).

*H. volcanii* encodes a family D polymerase PolD, consisting of subunits DP1 and DP2. The gene encoding the small exonuclease subunit DP1 (HVO_0003) is located in close proximity to *oriC1* and the gene encoding Orc1, while the gene for the large catalytic subunit DP2 (HVO_0065) is located distal to the origin. Both DP1 and DP2 are essential for PolD activity; the two subunits stimulate the activity of one another, as seen for *Pyrococcus* species [177]. Sequence and domain analyses show that DP1 and DP2 are similar to other euryarchaeal family D polymerase subunits, and DP2 contains a C-terminal PIP domain for interaction with PCNA [87]. Thus far, little work has been carried out into the function of PolD in *H. volcanii*, apart from establishing that PolD is essential for cell viability (T.A. 2020, unpublished).

##### 2.2.2.5. Single-stranded DNA-binding proteins

ssDNA-binding proteins (SSBs) play a central role in DNA replication, recombination and repair across all domains of life but share limited sequence conservation [188–193]. They function to coat ssDNA exposed during DNA replication, protecting it from degradation or chemical modification. In addition, SSBs can assist in homologous recombination by inhibiting secondary structure formation on ssDNA [194,195] or by interacting with RadA recombinase to promote strand exchange, as has been observed in the euryarchaeon *P. furiosus* [196]. Consistent with their role in DNA replication, SSBs are generally essential for viability in bacteria [197,198], eukaryotes [199,200] and archaea [201,202].

Bacteria use homotetrameric SSB, which forms nucleoprotein filaments along ssDNA. Eukaryotes bind ssDNA with hetero-trimeric replication protein A (RPA), which is structurally and functionally analogous to bacterial SSB [203]. Depending on species, the ssDNA-binding protein in archaea can be bacterial-like (SSB) or eukaryotic-like (RPA). For example, *S. solfataricus* uses a protein structurally akin to SSB [204], while *P. abyssi* encodes a heterotrimer showing homology to eukaryotic RPA [196]. A group of 10 species of Crenarchaea, belonging to the clade Thermoproteales, lack a canonical SSB; instead they encode a protein termed ThermoDBP that supplies the essential ssDNA-binding activity in the absence of SSB [205].

*H. volcanii* encodes three homologues of a eukaryotic-like SSB: RPA1 (HVO_1338), RPA2 (HVO_0519) and RPA3 (HVO_0292) [201]. All three RPA homologues contain OB folds that facilitate DNA binding, with each OB-fold consisting of five ß-sheet strands folded into a barrel-like structure. The binding of this barrel around ssDNA stabilizes the DNA and prevents attack by nucleases. Although structurally similar, each RPA protein has a unique function and they do not form a hetero-trimeric complex as seen in *P. abyssi* [196,206]. RPA2 is the only homologue essential for cellular survival, while RPA1 and RPA3 are both non-essential [201]. Formation of RPA2 foci has been seen in cells treated with aphidicolin, an inhibitor for PolB, indicating an essential role for RPA2 in overcoming replication stress [207,208]. RPA2 foci formation has also been observed in cells treated with ultraviolet (UV) light, suggesting an additional role for RPA2 in DNA repair.

Deletion of the gene encoding RPA1 results in no increase in sensitivity of cells to DNA damaging agents, indicating no major role in DNA repair [201]. RPA1 has been genetically linked with RPAP1 (RPA-associated protein 1; HVO_1337), an OB-fold protein predicted to assist RPA and facilitate ssDNA binding. RPA1 and RPAP1 are both located in the same operon with co-purification studies indicating an *in vivo* association between the proteins [209]. By contrast, cells deleted for RPA3 are sensitive to DNA damaging agents including UV radiation, phleomycin and methyl methane sulfonate (MMS). This indicates a role for RPA3 in DNA repair, in particular double-strand break (DSB) repair, as the aforementioned agents promote DSB formation [201]. Akin to RPA1, RPA3 is also encoded within an operon alongside an RPA-associated OB-fold protein, RPAP3 (HVO_0291). A similar increased sensitivity to multiple DNA damaging agents was seen upon the deletion of RPAP3 [209]. Whether this role in DNA repair also extrapolates to DNA replication is yet to be investigated; given that RPA3 is not essential, any role in DNA replication is likely to be minor.

RPA homologues present in the closely related species *Halobacterium* have also been implicated in DNA repair and deletion mutants show increased sensitivity to various DNA damaging agents [202]. *Halobacterium salinarium* possesses 5 SSB homologues (2 eukaryotic-like, 2 bacterial and one euryarchaea-specific). Upon deletion of these homologues, cells display increased sensitivity to infrared (IR) and UV radiation, and mitomycin C (MMC) treatment, with the strain deleted for the euryarchaeal-specific RPA homologue being most sensitive [202]. Despite the high degree of homology between these two halophiles, there are functional differences regarding DNA replication and repair mechanisms; *H. volcanii* RPA proteins are implicated only in DSB repair, while *Halobacterium* homologues appear to be playing a role in multiple DNA repair pathways [201,202,209].

##### 2.2.2.6. Other replisome components

Lagging strand maturation requires the removal of RNA primers on Okazaki fragments; the resulting gap is filled, and nicks are ligated to give a continuous DNA strand. RNase H proteins act to remove RNA primers associated with Okazaki fragments, flap endonucleases remove any flap structures generated in displacing primers, and DNA ligase seals any remaining nicks to give a complete product. In eukaryotes, gap filling is an early event, occurring prior to removal of the CMG complex from dsDNA [210].

The RNase H family of proteins acts to remove RNA primers from fully replicated Okazaki fragments; they also degrade R-loops (RNA-DNA hybrids) in a sequence-independent manner. RNase H enzymes are evolutionarily conserved and although not essential for cell survival, their deletion leads to strong sensitivity to DNA damaging agents in eukaryotes [211,212]. They can be categorized into three groups: RNase H1 proteins are present in bacteria, archaea and eukaryotes, and in reverse transcriptases from retroviruses and retroelements; RNase H2 proteins are present in all domains of life, usually together with a RNase H1 [213]; RNase H3 proteins are found in some bacteria and archaea, and show structural similarities to RNase H2 [213]. RNase H proteins generally share low sequence similarity, but both RNase H1 and RNase H2 group proteins use a highly conserved two metal ion catalytic mechanism [214,215]. All archaea encode an RNase H2 similar to the eukaryotic enzyme [213], together with an RNase H1 or RNase H3 [216].

*H. volcanii* possesses encodes five RNase H homologues, three of type 1 (RNase H-E, HVO_0732; RNase H-A, HVO_2438; RNase H-C, HVO_A0463) and a single type 2 protein (RNase H-B HVO_1978). RNase H-D (HVO_A0277) does not fit clearly into either group and its function remains unknown. Type 2 RNase H-B is non-essential in *H. volcanii*. It encodes a C-terminal PIP domain, implicating this RNase H at the replication fork. *In vitro* reconstitution of RNA primer removal in *P. abyssi* has implicated RNase H in cutting the RNA : DNA hybrid at Okazaki fragments as an early step, allowing subsequent strand displacement by PolB/PolD [217]. The roles of the three type 1 RNase H genes in *H. volcanii* remain unknown and warrant further study.

Fen1 (Flap endonuclease 1) is a structure-specific endonuclease that acts to remove 5′ overhangs generated during Okazaki fragment maturation as a result of strand displacement. Replicative DNA polymerases will then act on the newly generated 3′ end to fill the gap and DNA ligase will seal the nick. The eukaryotic polymerase responsible for final synthesis (gap filling) still remains a controversial topic. In both *Caenorhabditis elegans* and *Xenopus laevis*, Pol-ε, but not Pol-δ, has been shown to interact with the post-replication CMG complex [218]. DNA incorporation studies in *P. abyssi* have shown a reduced incorporation of nucleotides in the absence of Fen1, indicating a possible role for Fen1 in archaeal DNA replication [137].

*H. volcanii* encodes a single Fen1 homologue (HVO_2873), with deletion of this nuclease being viable [219]. This is in contrast with *Halobacterium* sp. NRC-1 where its single Fen1 homologue, *rad2*, is essential [172]. Rad2 has been implicated as a key player in UV damage repair in *Halobacterium* [220]; similarly, *H. volcanii* strains lacking Fen1 display increased sensitivity to UV and DNA cross-linking agents [219]. The fact that *fen1* can be deleted from *H. volcanii* suggests redundant systems are in place to deal with DNA damage in this species.

### 2.3. Termination of replication

Termination of DNA replication involves the convergence of two replication forks, either at random or at a defined location(s) depending on the organism, followed by dissociation of the replisome and decatenation of the chromosomes to allow correct segregation into daughter cells [221].

#### 2.3.1. Sites of termination

In bacteria, specific regions on the chromosome called termination (*Ter*) sites dictate where replication is halted, they are generally located at the furthest point from the origin. *Ter* sites act as a polar block to the DNA replication machinery, causing the replication fork(s) to stall within the defined termination region. Up to 10 ter, sites (named *TerA-J*) are bound by the DNA replication terminus-binding protein Tus in a specific orientation [222]. The replication fork is able to bypass 5 Tus-bound *Ter* sites with the *ter* sites terminating the replisome. Unlike bacteria, eukaryotes do not have sequence-defined termination sites. Termination events occur midway between two origins, with more active origins displaying more defined regions of termination [223–225]. Some studies have indicated the convergence of CMG complexes is a key step in the initiation of termination in eukaryotes [226].

While archaea share a circular genome architecture with bacteria, their chromosomes lack defined termination sites [60,227]. Instead, the termination of replication appears to occur in ‘zones’ where replication forks meet randomly, as in eukaryotes. This is visible on replication profiles (MFA plots), where termination zones map as broad valleys; this contrasts with the sharp ‘canyons’ seen for bacteria, which represent defined termination sites. Work carried out on *Sulfolobus* species has shown replication to be asynchronous, suggesting both number of origins and rate of initiation may affect where termination occurs [228].

Little is known about the details of termination of DNA replication in *H. volcanii*. The broad termination zones seen equidistant to origins of replication on MFA plots suggest that *H. volcanii* does not encode defined *ter* sites, and the relocation of such termination zones upon deletion of origins confirms there is no sequence specificity to termination in this species [60].

#### 2.3.2. Dissociation of the replisome

Prior to completion of replication, components of the replisome must be removed to prevent over-replication and to allow segregation of the newly synthesized DNA. During DNA synthesis in eukaryotes, the CMG replicative helicase complex encircles ssDNA, opening the helix to allow processive elongation. When converging with another fork, the CMG complex will bypass the CMG complex of the oncoming replisome and switch from binding ssDNA to binding dsDNA; the location of the CMG complex on the leading strand of both replisomes ensures there is no steric clash, and no decrease in synthesis rate is observed at termination sites in *Xenopus* [210]. The switching of binding of the CMG complex from ssDNA to dsDNA acts as a marker for downstream events. Polyubiquitylation of MCM subunit MCM7 by specific E3 ligases leads to unloading of the CMG complex by the activity of ATPase Cdc48/p97 [229,230]. Dissociation of MCM from the heart of the replisome is hypothesized to cause dissociation of the entire replisome. However, some predictions have been made that it is the unloading of PCNA, and its numerous associated proteins, which results in an unloading of further replicative factors. Such a model for coordinating termination of replication has been proposed in archaea for *S. solfataricus*, whereby PCNA coordinates the termination activity of PolB1, flap endonuclease 1 (Fen1) and DNA ligases, due to the presence of PIP domains on these proteins [231].

Re-replication events are not seen in eukaryotes, indicating the presence of strict regulatory mechanisms in termination; the use of ubiquitylation adds a layer of complexity to eukaryotic termination, which is not seen for bacteria [232]. Little is understood regarding the removal of replication components in archaeal species, including *H. volcanii*, but preliminary evidence suggests a system more complex than that of bacteria. It remains to be elucidated if post-translational modifications play a role in archaeal termination and replisome unloading, but given that homologues of Cdc48/p97 and ubiquitin-like proteins are both found in *H. volcanii* [233], this remains a distinct possibility.

#### 2.3.3. Decatenation and resolution

Unwinding of DNA during replication will lead to overwinding of the duplex ahead of the replication fork, forming supercoils. If left unresolved during replication, this increased torsional stress would prevent the replication fork from proceeding along with the duplex and at termination would prevent equal segregation of DNA to daughter cells.

In bacteria, topoisomerases act to control the level of torsion in DNA during replication [234]. Type II topoisomerases are important in termination: DNA gyrase acts to relieve positive supercoils formed as a product of DNA unwinding while TopoIV resolves pre-catenanes, allowing fork convergence to occur and to be resolved successfully [235,236]. Following the resolution of torsional stress, RecG translocase and RecBCD helicase-nuclease resolve overlapping sequences at the terminus, giving a product suitable for dissolution and segregation [237,238]. Should an odd number of crossover events occur, chromosome dimers can be created [239]. Such structures must be separated prior to segregation to ensure each daughter cell receives a full genome complement. In bacteria, Xer site-specific recombinases act at specific loci named *Dif* (differential induced filamentation) sites, which are in close proximity to *ter* sites, and resolve chromosome dimers into monomers [240].

Argonaute family proteins (AGO) are found across all domains of life. In eukaryotes, short RNA guides act to target AGO proteins against transposons and viruses while in bacteria, AGO proteins have been shown to defend against transformation by DNA plasmids. Recent work has implicated Argonaute protein in termination and decatenation of DNA replication in the bacterium *T. thermophilus* [241]. When DNA gyrase is inhibited, Argonaute is capable of completing DNA synthesis and ensuring correct decatenation of the chromosome [241]. In the absence of both DNA gyrase and Argonaute activities, chromosome resolution does not occur [241]. Such a critical function for AGO proteins in DNA replication has not previously been observed, and further work is warranted to see if AGO proteins act in chromosome resolution in archaea.

For eukaryotes, the completion of DNA replication will lead to daughter molecules that are catenated to one another. Any pre-catenanes present would also be converted to full catenanes for processing [242]. The specific details of resolution in eukaryotes remain unknown; however it is believed that topoisomerase II (TopoII; type II topoisomerase) is essential for the process [243,244]—inactivation of TopoII leads to stalling in G2 phase, resulting in a build-up of catenanes and failure to complete replication [245].

Although no *Ter* sites or Tus homologues have been identified in archaea, homologues of *Dif* and Xer have been identified [68,227,246]. Some archaeal species (e.g. *Thermococcus*) possess *Dif* sequences at zones of termination, suggesting coordination of chromosome monomerization and replication termination [246]. However, in *Sulfolobales*, *Dif* sites are situated far away from termination zones, suggesting that these two processes may be less tightly coupled [227].

*H. volcanii* possesses multiple XerC/D-like homologues, suggesting the possibility of *Dif* sites. Of the 12 *xerC/D* genes, four have been deleted without impacting viability (HVO_1422, deleted by Uri Gophna, HVO_2259, HVO_2273 and HVO_2290 deleted by T.A.; T.A. 2020, unpublished). Whether these XerC/D-like enzymes have a role in the termination of replication remains to be determined. The presence of broad zones of termination coupled with the presence of *Dif* sites hints at archaea carrying both bacterial- and eukaryotic-like mechanisms of chromosome resolution and termination.

Topological stress in *H. volcanii* can be imagined to be a large problem. There are approximately 20 genome copies within each cell, which are replicated asynchronously due to the lack of a defined cell cycle. Relieving superhelical torsion is carried out by the action of three topoisomerases: DNA topoisomerase IA (TopoIA; HVO_0681), DNA topoisomerase VI comprising subunit A (HVO_1570) and B (HVO_1571), and DNA gyrase comprising subunit A (HVO_1572) and B (HVO_1573). The laboratory strain of *H. volcanii* displays sensitivity towards novobiocin, an inhibitor of DNA gyrase, indicating that DNA gyrase is essential for viability [247,248]. Both subunits of DNA TopoVI have also been shown to be essential (T.A. 2020, unpublished). Further work is needed to assess the interplay of the different topoisomerases in *H. volcanii* and how they act together to resolve chromosome structures for segregation.

Following decatenation, RNA primers on Okazaki fragments are removed; the resulting gap is filled by replicative DNA polymerases, and nicks are ligated to give a continuous DNA strand. RNase H proteins act to remove RNA primers associated with Okazaki fragments; flap endonucleases remove any flap structures generated in displacing primers, and ATP-dependent (and in some species, NAD-dependent) DNA ligases (to be discussed in detail later) seals any remaining nicks to give a complete product. In eukaryotes, gap filling is an early event, occurring prior to removal of the CMG complex from dsDNA [210].

### 2.4. DNA repair

Environmental and endogenous factors threaten the genome integrity of all living organisms. Damage to DNA can lead to mutagenesis, genome instability, senescence and cell death [249]. The majority of DNA damage lesions arise from endogenous sources during normal cellular metabolic processes, generating oxidation, hydrolysis and alkylation damage, along with the insertion of mismatched DNA bases. Environmental agents such as UV light, ionizing radiation and various chemical agents generate base lesions including the deamination of cytosines, adenines and guanines, depurination of bases, oxidative damage, as well as DNA double-strand breaks (DSBs) [249–253].

While evolution is driven by rare advantageous mutations, efficient DNA repair is a requirement of all forms of life as large amounts of unrepaired damage cannot be tolerated. This is especially true for many archaeal species, which inhabit demanding environments. Extremes of salinity, temperature and pH can increase the load of DNA damage faced by these organisms and thus they require robust methods of repair. A recent study has estimated the genome-wide mutation rate and spectrum in *H. volcanii*; the base substitution rate of 3.15 × 10^−10^ per site per generation is similar to that seen in mesophilic species [254].

Cells have developed a plethora of DNA repair mechanisms, and generally, these repair mechanisms differ depending on the type of DNA damage incurred [255]. While a small number of specific chemical modifications can be repaired by a single protein without a requirement for cutting of the DNA backbone, a process known as direct repair, mismatched and damaged bases are more commonly repaired by one of four excision pathways: MMR, BER, nucleotide excision repair (NER) and ribonucleotide excision repair (RER) (table 1).

One of the most harmful DNA lesions is the DSB, since it affects both strands of the duplex. If unrepaired, DSBs can give rise to large-scale genome rearrangements, chromosomal translocations, significant mutagenesis and cell death. Due to the potential danger of DSBs, the most complex DNA repair mechanisms are responsible for repairing this type of DNA damage. The major DSB repair pathways are HR, non-homologous end joining (NHEJ) and microhomology-mediated end joining (MMEJ). HR is a high-fidelity mechanism that generates error-free products, while NHEJ and MMEJ pathways are quicker but error-prone processes, which can result in deletions, insertions and translocations [256–260].

Generally, DNA repair processes are highly conserved throughout evolution. Archaea share many components of their DNA repair machinery with eukaryotes [253,261], but halophilic archaea also carry numerous enzymes that have been acquired by LGT from bacteria. Furthermore, halophilic archaea, which inhabit UV-intense hypersaline environments, have gained further strategies to prevent damage. Polyploidy provides an evolutionary strategy for DNA damage resistance [65,262], an option that is not usually available in organisms like *Saccharomyces cerevisiae* [263], and many halophiles use photoprotective membrane-associated pigments such as carotenoids [264].

### 2.5. Direct repair

#### 2.5.1. Photolyases

Halophilic archaea experience a significant dose of UV light in their natural environments and need efficient mechanisms to repair UV-induced DNA damage. Photoreactivation is a light-dependent direct repair mechanism catalysed by photolyase. In a single step, photolyase is able to reverse cyclobutene pyrimidine dimer (CPD) and 6–4 pyrimidine-pyrimidine photoproduct (6–4PP) lesions formed as a consequence of solar UV radiation [265,266]. Photoreactivation is the most important repair mechanism for surviving high doses of UV light in nature [267].

Photolyases are present in several archaea and share homology with those present in bacteria and eukaryotes, suggesting that this mechanism arose early during evolution [255,268,269]*.* Few archaeal species encode more than one photolyase homologue; *Halobacterium* species are known to encode two as a result of a gene duplication event, encoded by genes *phr1* and *phr2* [267]. *H. volcanii* also encodes two photolyase homologues, *phr1* and *phr2* (HVO_2911 and HVO_2843 respectively)*,* alongside an uncharacterized photolyase-related gene *phr3* (HVO_1234). The requirement for more than one photolyase in these halophiles may be a consequence of the high amounts of UV damage experienced in their environment. However, genetic studies have shown not all *phr* homologues within a species are active, suggesting some redundancy [270].

#### 2.5.2. Methyltransferases

Restriction-modification (RM) systems have evolved to protect cells from invading DNA; RM systems comprise restriction endonucleases (RE) and DNA methyltransferases (MTases). RM systems are present in bacteria and archaea and allow the cell to differentiate between its own methylated DNA and foreign unmethylated DNA, which can be recognized and digested by the RE [271,272]. *H. volcanii* encodes a putative type IV Mrr RE (HVO_0682), which cleaves DNA that is methylated at GA^m6^TC sites; this includes Dam-methylated plasmid DNA extracted from *Escherichia coli*, hindering transformation protocols. Gene deletions of Mrr RE have been carried out to resolve such limitations [36].

MTases encoded in the absence of a cognate RE, known as orphans MTases, play essential roles in cellular processes such as DNA replication, DNA repair and gene expression [273]. These defence mechanisms have been extensively characterized in bacteria but are only poorly defined in archaea. The use of deletion mutants of genes predicted to be methyltransferases in combination with single-molecule real-time (SMRT) sequencing has allowed the detection and mapping of methylated bases throughout the genome [274]. Two methylated motifs were detected in the *H. volcanii* genome: C^m4^TAG and GCA^m6^BN_6_VTGC (where B stands for C, G or T, V stands for A, C or G, and N stands for any base). Genes responsible for DNA methylation in *H. volcanii* include HVO_A0006, HVO_0794 and HVO_A0237 that methylate cytosine at C^m4^TAG, and the type I RM operon *rmeRMS* (HVO_2269–2271) that methylate adenine at GCA^m6^BN_6_VTGC motifs [275].

### 2.6. Excision repair

#### 2.6.1. Base excision repair

**Figure 2.**
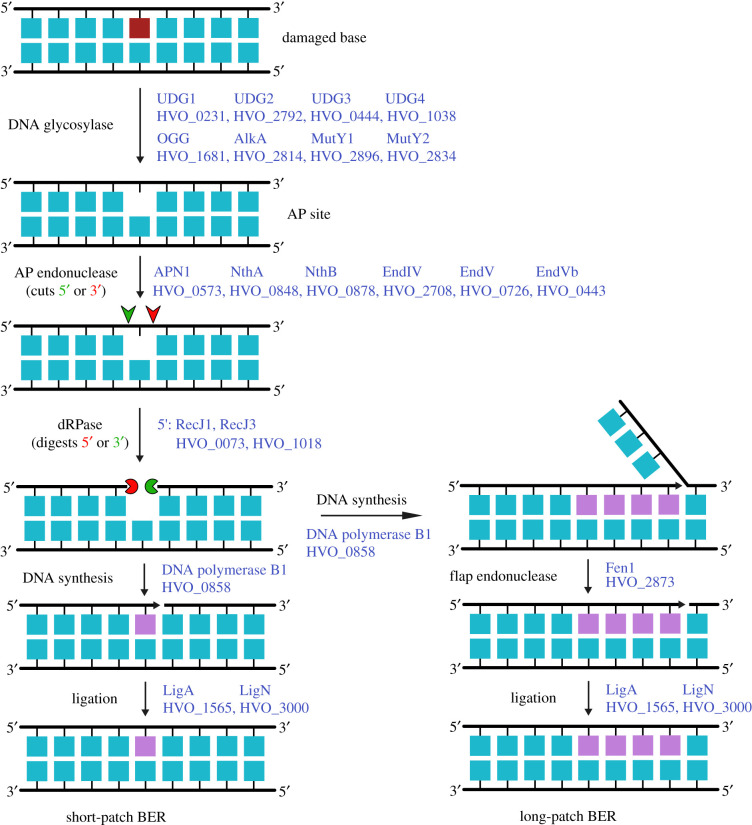
Base excision repair. The damaged base (red) is recognized and removed by DNA glycosylases, which cleave the N-glycosyl bond between the damaged base and the sugar to generate an apurinic or apyrimidinic (AP) site. AP endonucleases cleave 5′ of the abasic site or ß lyase cleaves 3′ of the site, and the backbone is removed by phosphodiesterases. Short-patch BER uses DNA polymerases (PolB1; HVO_0858 in *H. volcanii*) to insert the missing nucleotide (purple) with DNA ligases (LigA; HVO_1565 or LigN; HVO_3000) linking the newly synthesized nucleotide to the sugar backbone. In long-patch BER, DNA polymerases insert 2–6 nucleotides at the gap to generate a flap structure. Flap endonuclease Fen1 (HVO_2873) cleaves the displaced strand and DNA ligases seal the DNA backbone.

Base excision repair is conserved across all domains of life (figure 2 and table 1). The canonical pathway involves the action of a DNA glycosylase specific for a damaged base, which cleaves the N-glycosyl bond between the damaged base and the sugar, generating an apurinic or apyrimidinic (AP) site. Most DNA glycosylases are mono-functional but some are bi-functional with a coupled β-lyase activity that cleaves 3′ of the AP site by β-elimination. Alternatively, the AP site is cleaved on the 5′ side by an AP endonuclease that acts by hydrolysis. The AP product is then degraded by a 3′ or 5′ phosphodiesterase, respectively, leaving a single-nucleotide gap with a 3′ hydroxyl and either a 5′ deoxyribose phosphate (5′dRP) or a 5′ phosphate. The generation of the 5′ end allows a family X DNA repair polymerase (Pol-β in eukaryotes) to begin synthesis, filling the gap. While 5′ phosphate can only be repaired by short-patch repair, 5′dRP can be repaired by both short- and long-patch repair [276]. Short-patch repair is where the family X polymerase, Pol-β, synthesizes the single nucleotide, removing the 5′dRP with its inherent lyase activity. Long-patch repair is more complex and involves the insertion of a further 4–6 nucleotides to generate a flap structure that displaces oncoming DNA, which is subsequently removed by flap endonuclease (FEN-1 in eukaryotes) [277,278]. While long-patch repair still uses Pol-β, family B DNA polymerases Pol-δ and Pol-ε have also been implicated in this mechanism of BER.

In some archaea, evidence supports the family B polymerase PolB as the candidate for BER synthesis [276]. Among replicative DNA polymerases, PolB has been described to be involved in DNA repair, but not the archaea-specific family D polymerase PolD [167,170]. In contrast with eukaryotes, few family X DNA polymerases have been identified in archaea thus far, suggesting an alternative method of BER [279]. In archaea, Fen1 is implicated in the removal of RNA primers from Okazaki fragments during lagging strand DNA replication and Fen1 orthologues have been described in both Euryarchaeota and Crenarchaeota [280–284]. To date, only Fen1 from *M. thermautrophicus* has been shown to be actively involved in BER [285]. The Rad2-family flap endonuclease is essential in *Halobacterium sp*. NRC-1 [172], potentially offering an alternate flap endonuclease used by archaea for resolution of long-patch repair.

Both long- and short-patch BER result in a nick that is targeted for ligation by a DNA ligase. Bacterial and eukaryotic ligases show no homology. While bacterial ligases are NAD dependent, eukaryotic ligases are ATP dependent. Most archaea possess eukaryotic-like ATP-dependent DNA ligases [286] but haloarchaeal species can encode more than one type of DNA ligase, including bacterial-like NAD-dependent ligases.

In *H. volcanii*, in addition to the ATP-dependent ligase LigA (HVO_1565), an NAD-dependent ligase LigN (HVO_3000) is present. Halophilic species are known to undergo large amounts of gene transfer, thus it is likely this NAD-dependent ligase has been acquired by LGT from bacteria [287,288]. Neither DNA ligase alone is essential, but a double mutant is inviable, indicating that the two ligases are redundant for their essential function; most likely the ligation of Okazaki fragments during lagging strand DNA synthesis. *H. volcanii* strains lacking eukaryotic-like ligase *ligA* show higher UV sensitivity compared with wild type, indicating that this enzyme could play a role in DNA damage resolution in BER and/or NER [288,289].

An alternative excision repair (AER) pathway has been described which is initiated by an endonuclease rather than a glycosylase [290]. Two endonucleases able to nick damage-containing DNA have been characterized: Endonuclease V (EndoV) and Endonuclease Q (EndoQ). EndoQ cuts 5′ of uracil, hypoxanthine and abasic sites [291,292], while EndoV shows a preference for cutting 3′ of lesions [293]. EndoV is widely conserved in bacteria, eukarya and archaea [294], while EndoQ has a much narrower distribution throughout the three domains [292]. Within archaea, EndoQ is found within a subset of the phylum Euryarchaeota, including *Thermococcales* and numerous methanogenic orders, and is often found in combination with EndoV. This is in contrast with the majority of crenarchaeal and halophilic species, including *H. volcanii*, which encode only EndoV (HVO_0726). EndoQ from *T. kodakarensis* and *P. furiosus* has been shown to be stimulated by interaction with PCNA *in vitro* [295], which may coordinate its action at the replication fork.

#### 2.6.2. Nucleotide excision repair

Nucleotide excision repair is a DNA repair mechanism that recognizes and removes a large number of different helix-distorting lesions [296,297]. Examples of NER substrates are CPDs and 6–4PPs generated by UV radiation, reactive oxygen species (ROS)-induced base modifications or base adducts created by exogenous chemical agents. NER is the primary mechanism to repair UV-induced DNA lesions in the absence of photoreactivation; because NER is light-independent, it is often referred to as ‘dark repair’ [298]. The basic steps of the process are conserved in all domains of life, but bacterial and eukaryotic NER proteins show very little homology.

The NER pathway in bacteria is catalysed by the UvrABC excision repair machinery: UvrA is responsible for damage recognition, UvrB helicase separates the two DNA strands and UvrC nuclease cuts at both sides of the lesion. Primarily, UvrA : UvrB will scan DNA, with the UvrA subunit recognizing bulky lesions in the template. Upon recognition, UvrA dissociates and UvrC binds UvrB (giving UvrBC). UvrBC will act to cleave up- and downstream of the lesion. UvrD is a superfamily I helicase member and moves in a 3′–5′ direction [296], acting to peel away the excised segment containing the damaged DNA. This permits access to family A polymerase DNA Pol I and DNA ligase, which act to fill and seal the gap, respectively. A small number of archaeal species carry homologues of the bacterial NER proteins; they are primarily found in mesophilic methanogens and halophiles [219,299].

Alongside global genomic NER (GG-NER), transcription-coupled NER (also known as transcription-coupled repair, TCR) forms a sub-pathway of NER. TCR functions to remove RNA polymerase-arresting DNA lesions from the template of actively transcribed genes [300]. Usually, repair by TCR is quicker than that of canonical NER, thus favouring correction of lesions within the transcribed strand of DNA [301]. TCR is initiated when RNA polymerase stalls at a lesion in the transcribed strand of DNA. In bacteria, RNA polymerase is displaced by NER proteins, which are recruited to the site of damage by a transcription-repair coupling factor (TRCF), otherwise known as Mfd protein [300,302].

Eukaryotes use a more complex pathway encoded by 9 proteins (named XPA to XPG). While differing in complexity, eukaryotic NER follows the same general principle as bacterial NER. Akin to bacteria, eukaryotic NER can be split into two sub-pathways: GG-NER and TCR. GG-NER can occur throughout the genome, while TCR is responsible for timely repair of lesions in the transcribed strand of active genes. In eukaryotic GG-NER, damage recognition is carried out by XPC-Rad23B (or by DDB1/2 in heterochromatin) and the DNA is opened by transcription factor IIH (TFIIH), a multi-protein complex containing helicase subunits XPB and XPD [303]. Following opening, binding of XPA and SSB protein RPA results in the recruitment of nucleases ERCC1-XPF and XPG to cleave either side of the lesion (dual incision). Canonical family B replicative DNA polymerases Pol-δ and Pol-ε, along with error-prone family Y translesion synthesis polymerase Pol-κ, have been implicated in gap filling [304], with requirements having been shown to change depending on cell cycle stage [305]. The same applies to the type of DNA ligase used for sealing the nick; DNA ligase III*α* and DNA ligase I have both been linked to NER in eukaryotes [305,306]. In eukaryotic TCR, the stalled RNA polymerase itself acts as the signal for recruitment of NER proteins; the stalled polymerase works in the place of XPC-Rad23B, leading to recruitment of downstream components as previously described.

**Figure 3.**
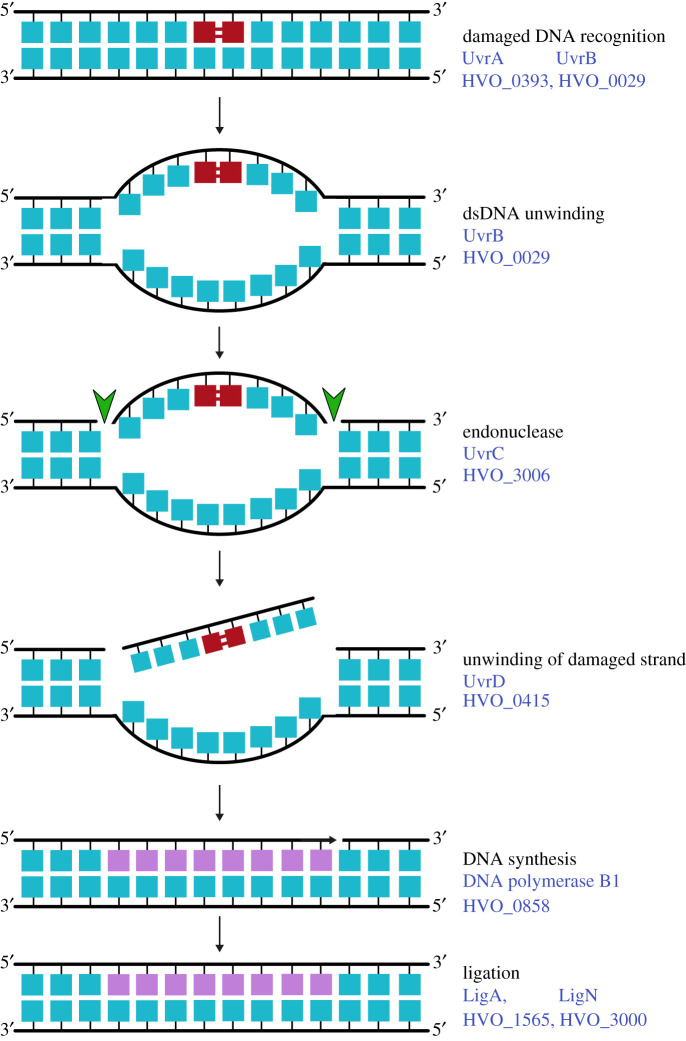
Nucleotide excision repair. In *H. volcanii,* bulky DNA adducts (red) are recognized by UvrA (HVO_0393). UvrB (HVO_0029) initiates DNA unwinding around the damage site through its helicase activity. Incisions 5′ and 3′ to the damaged bases are carried out by the endonuclease UvrC (HVO_3006). Unwinding of the damaged strand is carried out by a helicase such as UvrD (HVO_0415). The remaining gap is filled (purple) by replicative DNA polymerase PolB1 (HVO_0858) and the newly synthesized DNA is attached to the backbone by the activity of DNA ligases (LigA; HVO_1565 or LigN; HVO_3000).

*H. volcanii* carries homologues of UvrABC proteins, encoded by genes *uvrA* (HVO_0393)*, uvrB* (HVO_0029) and *uvrC* (HVO_3006) (figure 3 and table 1) [35]. The bacterial NER genes are non-essential in *H. volcanii* and deletion mutants in *uvrABC* show enhanced sensitivity to UV damage in the absence of photo-reactivating light [219], implicating UvrA, UvrB and UvrC in ‘dark repair’ of UV lesions in *H. volcanii*. Cells deficient in UvrD (HVO_0415) show no such sensitivity phenotype and it has been proposed that a redundant helicase may substitute for UvrD [219]. Furthermore, *uvrABC* mutants, but not *uvrD*, exhibit increased sensitivity to the DNA inter-strand cross-linking agent MMC [307]. Similar results have been shown for other haloarchaeal species, including *Halobacterium* sp. NRC-1 [265] where deletion of *uvrABC* genes results in UV sensitivity, indicating that the bacterial Uvr system is required for the repair of UV-induced DNA damage. Intriguingly, *Halobacterium* also encodes homologues of eukaryotic-like NER proteins XPB and XPD (helicases), as well as XPF (endonuclease).

Genetic experiments in *H. volcanii* have revealed a link between the bacterial NER protein UvrA and NreA (HVO_0734), a member of the archaea-specific Nre family of proteins [307]. Most archaea encode at least one Nre homologue with a C-terminal PIP motif, while some species encode a second protein, NreB, with a less well-defined PIP motif [308]. *H. volcanii* encodes only NreA, which is not essential but cells lacking NreA are sensitive to MMC treatment. Double deletion mutants of *nreA* and *uvrA* are no more sensitive than single mutants, suggesting NreA and UvrABC act in the same pathway in *H. volcanii* [307]. NreA has been proposed to be involved in the repair of MMC-induced DSBs, acting in combination with the UvrABC NER system, and the interaction of NreA and PCNA has been shown to be essential for this role [307].

The majority of archaeal species, including hyperthermophiles, carry homologues of eukaryotic-like NER proteins, including endonucleases XPF/XPG and helicases XPB/XPD [253,289,309–313]. *S. islandicus* and *T. kodakarensis* mutants lacking XPB and XPD helicases are only slightly sensitive to UV radiation, MMC and MMS treatments, indicating that these enzymes do not play an essential role in NER [111,314]. Some archaea carry both bacterial UvrABC-like proteins and an incomplete eukaryotic XP system, for example *Methanosarcina mazei* [289] and various halophilic species [255]. As previously discussed, *H. volcanii* encodes homologues of the bacterial Uvr system. However, it also encodes homologues of endonucleases XPF (Hef, HVO_3010) and XPG (Fen1, HVO_2873) [35]. Unlike XPF in eukaryotes, Hef in *H. volcanii* has been shown to play no role in NER, instead being implicated in the restart of stalled replication forks [219].

TCR, while observed in some archaeal species, does not seem to be universally conserved throughout the domain. The crenarchaeon *S. solfataricus* does not favour repair of transcribed strands suggesting that the system for TCR in this species (if present) is no faster than that of GG-NER [315,316]. However, the RNAP of euryarchaeon *T. kodakarensis* has been shown to pause at a variety of DNA lesions suggesting damage recognition by the RNAP itself, akin to mechanisms of TCR in other domains of life [317]. To date, no homologues of bacterial TRCF have been identified in archaea [255]. While TCR is known to occur in halophilic species, the specifics of the mechanism remain unknown. In *H. volcanii*, TCR has been shown to occur in the absence of UvrA, unlike *Halobacterium sp.* NRC-1, which is unable to efficiently repair UV damage without UvrA [318]. In the latter species, UvrA protein seems essential for the initial recognition of the DNA damage in both GG-NER and TCR. By contrast, the initial recognition event for TCR in *H. volcanii* is UvrA independent; this could be performed by the RNA polymerase itself (as seen in eukaryotes and *T. kodakarensis*) or by an as yet unidentified coupling factor [318,319].

#### 2.6.3. Mismatch repair

The MMR machinery is conserved across bacteria and eukaryotes, but most archaea lack key components of the canonical pathway [255]. In bacteria and eukaryotes, the canonical MutS-MutL MMR pathway is able to detect and repair mismatched nucleotides that arise as a consequence of misincorporation by DNA polymerases [320]. MutS or MutL homologues are absent from many archaeal species [313,321], but are found in halophiles, methanogens and a few other euryarchaea; these species are all subject to LGT and their *mutSL genes* are thought to have been acquired from bacteria [322–325]. Archaea lacking MutSL proteins show a rate of spontaneous mutation similar to organisms that possess MutSL [321,326], indicating the presence of an alternative MMR mechanism. Furthermore, the deletion of *mutS* and *mutL* genes in *Halobacterium* sp. NRC-1 does not increase the mutation rate [327], suggesting that some archaea may carry more than one active MMR system.

EndoMS (also named NucS) is an endonuclease first characterized *in vitro* in *P. abyssi*, where it acts on branched DNA structures containing flapped and splayed DNA [328–331]. EndoMS is present in bacteria of the phylum Actinobacteria and in most members of archaea that lack functional MutSL homologues. EndoMS was initially proposed as a potential NER endonuclease due to its ability to cleave flapped and splayed structures, akin to XPF [312]. Both *S. solfataricus* XPF and *P. abyssi* and *T. kodakarensis* EndoMS/NucS are able to interact with PCNA, and this interaction is required for their specificity and endonuclease activity [329,330,332,333]. PCNA may also assist EndoMS and XPF to access the site of damage and facilitate the following steps of the repair by enabling interactions with other repair proteins.

Ishino *et al.* [333] have demonstrated that *T. kodakarensis* EndoMS has the ability to specifically cleave dsDNA containing a base pair mismatch *in vitro*, indicating a role for EndoMS in a novel MMR pathway. Other structural studies have supported the hypothesis of EndoMS specifically recognizing and binding mismatched bases by a unique dual base flipping mechanism [334,335]. In *T. kodakarensis*, the *endoMS* gene is in an operon with the *radA* recombinase gene [329], but this genetic link is only conserved within the genus *Thermococcus.* Nevertheless, a functional link between EndoMS and HR has been observed in *Sulfolobus acidocaldarius*, where EndoMS has been shown to act in HR-mediated stalled fork repair to remove helix-distorting DNA lesions, overlapping with the role of XPF and suggesting a potential role for EndoMS in NER [336]. A new function for NucS was recently described in *T. gammatolerans*, where NucS is capable of cleaving uracil- and hypoxanthine-containing dsDNA, indicating an alternative to BER for the repair of deaminated bases in this species [337].

Interestingly, some haloarchaeal species encode both NucS and MutSL homologues, including *H. volcanii*; it encodes four MutS (*mutS1a* HVO_1940; *mutS1b* HVO_0552; *mutS5a* HVO_0191; *mutS5b* HVO_1354), two MutL (*mutLa* HVO_1939; *mutLb* HVO_0551) and one NucS protein (HVO_0486). NucS is not essential and no phenotype is observed in cells lacking *nucS* [330]. Two of the four *mutS* genes (namely *mutS1a* and *mutS1b*) are located in operons with a *mutL* partner and are predicted to function in MMR [35]; deletion of these canonical *mutSL* genes leads to an increase in the spontaneous mutation rate of *H. volcanii* (T.A. 2020, unpublished). The other two MutS proteins belong to a subfamily that does not seem to be involved in DNA repair in other organisms [255,338]. The possible interplay between MutS-MutL and NucS pathways in *H. volcanii* remains to be elucidated.

#### 2.6.4. Ribonucleotide excision repair

Ribonucleotides (rNMPs) that are misincorporated into genomic DNA are recognized and removed by the RER pathway. Archaeal D family DNA polymerases have been shown to incorporate 1 rNTP every approximately 1000 bases, and archaeal B family DNA polymerases every approximately 2500 bases [339,340]. RNase H2 creates a nick 5′ to the misincorporated rNMP. The 3′ end of this gap is displaced by DNA polymerases and cleaved by Fen1. The remaining gap is then sealed by the activity of DNA ligases, mirroring the mechanism for Okazaki fragment maturation. In *T. kodakarensis*, this pathway has been identified using computational methodology [340]. The activity of RNase H2 proteins, Fen1 and DNA polymerases in RER in *H. volcanii* remains to be demonstrated.

### 2.7. Translesion synthesis

Family Y DNA polymerases have a specialized function, whereby they are able to bypass various forms of DNA damage that block DNA synthesis by canonical replicative polymerases; this process is known as translesion synthesis (TLS) [341]. Family Y DNA polymerases are conserved across the three domains of life [342]. They have a larger and more accommodating active site with smaller thumb and fingers domains, which make little or no close or specific contacts with the nascent base pair [343]. The closed conformation of the fingers domain suggests that the canonical ‘induced-fit’ mechanism to ensure correct Watson–Crick base pairing does not take place [344]. Moreover, TLS polymerases do not have an exonuclease domain or any proofreading activity. Instead, these error-prone enzymes carry out low-fidelity DNA synthesis, with the aim of bypassing the lesion without halting the replication fork. On undamaged DNA, TLS polymerases incorporate an incorrect nucleotide once every 10^−1^–10^−3^ bases [341,345,346]. Some translesion polymerases are better than others at incorporating the correct base opposite particular DNA lesions, suggesting an element of specificity depending on the species [347]. Thus, the product of TLS can be error-free or error-prone, depending of the type of lesion encountered.

Most eukaryotes encode four-family Y DNA polymerases, each with specificity for different types of the lesion [348,349]. Meanwhile, bacteria and archaea generally only encode one or two TLS polymerases, each with a broader lesion specificity than their eukaryotic counterpart. Due to the error-prone nature of TLS, this pathway is a potential source of genome instability and thus requires tight regulation. In eukaryotes, this is controlled by post-translational modification of both PCNA and family Y DNA polymerases [350]. All eukaryotic family Y DNA polymerases contain PIP motifs, which facilitate the interaction with PCNA [349].

The properties, roles and functions of archaeal family Y DNAPs are not well understood, since most have been tested *in vitro* under non-physiological temperatures. The TLS polymerases found in archaea belong to the DinB subfamily of family Y DNAPs. This subfamily is the most diverse and is found throughout all domains of life. DinB polymerases are prone to making single-nucleotide deletions *in vivo* and *in vitro*, caused by template slippage where repetitive sequences are present [343,351,352].

Hyperthermophilic archaea encode only one family Y DNA polymerase. Depending on strain, *S. solfataricus* encodes either Dpo4 (polymerase IV) or Dbh (DinB homologue) [343,344,353–355]. All archaeal DinB-like polymerases studied to date are capable of replicating past abasic sites, and in the case of Dbh, incorporating dATP [344,356]. Dpo4 can bypass a UV-induced *cis-syn* cyclobutene thymine dimer and 8-oxo guanines with relative efficiency, compared with other family Y DNA polymerases, since it can accommodate two adjacent template bases into its active site [355,357]; similar observations have been made in yeast [358] and humans [359]. This feature is not observed for other DinB-like polymerases [360–363]. Similar to human family Y DNA polymerases, Dpo4 shows limited activity on G4 structures [364,365].

Until now, only family Y polymerases were believed to act in TLS, but recent work in *S. islandicus* has implicated a family B enzyme, Dpo2, in TLS repair of helix-distorting lesions [366]. The potential TLS activity of Dpo2 may compensate for the lack of a canonical NER pathway in this crenarchaeal lineage [253]. In *S. solfataricus*, a complex has been described where both family Y and family B polymerases are bound to PCNA and DNA [367]. This is consistent with the ‘tool-belt’ model proposed for bacteria [368] and eukaryotes [369]. Tethering to PCNA allows for the coordination of DNA synthesis and TLS activities at the replication fork, with rapid switching from the canonical polymerase to TLS enzyme at the site of damage, and vice versa upon bypass.

*H. volcanii* encodes one family Y polymerase, PolY (HVO_1302), which shares homology with bacterial DinB. As in eukaryotic family Y polymerases, *H. volcanii* PolY encodes a C-terminal PIP motif, but it has diverged somewhat from the canonical sequence [136]. Degeneracy of the PIP motif has been observed in other family Y polymerases, including eukaryotic proteins [370]. Deletion of *polY* is possible in *H. volcanii* (T.A. 2020*,* unpublished), suggesting that other pathways are available for the repair or bypass of bulky lesions that block DNA synthesis by canonical polymerases.

### 2.8. Double-strand break repair

**Figure 4.**
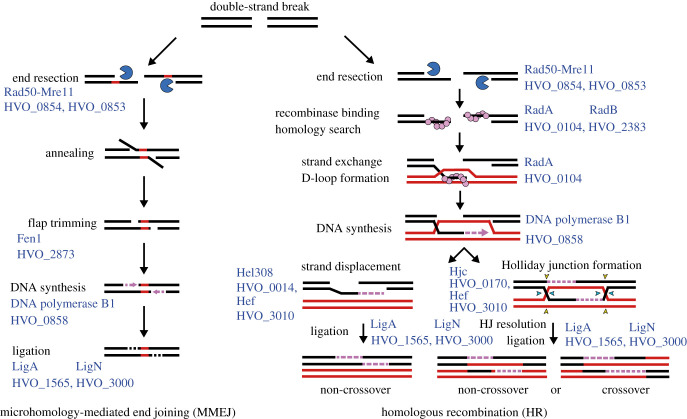
Double-strand break repair. Double-strand break repair in *H. volcanii* occurs by either microhomology-mediated end joining (MMEJ) or homologous recombination (HR). **MMEJ** begins with end resection at the break site which is carried out by Rad50 (HVO_0854) and Mre11 (HVO_0853). Homologous DNA strands around the break (red) is annealed and any displaced DNA is trimmed by Fen1 (HVO_2873). Gaps in both strands are filled (purple) by replicative DNA polymerase PolB1 (HVO_0858) and ligated by DNA ligases; in *H. volcanii* either LigA (HVO_1565) or LigN (HVO_3000). **HR** also involves initial end resection at the break site by the combined activities of Rad50 and Mre11. The single-stranded DNA ends are bound by the recombinase RadA (HVO_0104), aided by the recombination mediator RadB (HVO_2383), which then promotes the search for homologous DNA duplex (red). Strand exchange with the homologous duplex generates a D-loop (displacement loop) with a 3′ invading end, from which DNA is synthesized (purple) by the action of replicative polymerase PolB1 (HVO_0858). At this stage, either a non-crossover event occurs due to displacement of the invading strand by helicases such as Hel308 (HVO_0014) or Hef (HVO_3010); the free strand anneals with and is ligated to the other end of the DNA break using either LigA (HVO_1565) or LigN (HVO_3000). Alternatively, a Holliday junction is formed that is processed by resolvases Hjc (HVO_0170) or Hef (HVO_3010) to cut the four-way DNA junction, leading to either a crossover or non-crossover event between recombining chromosomes. The remaining nicks in DNA are resolved by either LigA or LigN.

DSBs are considered to be one of the most critical forms of DNA damage that cells can suffer. A break in both strands of the DNA double helix can lead to inhibition of key processes including DNA replication, transcription and cell division, alongside major genome rearrangements. Therefore, the major pathways of double-strand break repair (DSBR) are crucial for maintaining genome stability. The most accurate form of DSBR is homologous recombination (HR), but this is a complex and energetically costly process, and therefore less-demanding pathways of DSBR operate alongside HR (figure 4 and table 1).

#### 2.8.1. Non-homologous end joining

Classical non-homologous end joining (NHEJ) repairs DSBs by ligating DNA ends using little or no complementary base pairing. The first step is recognition of the DSB by Ku protein, comprising a Ku homodimer in bacteria and a Ku70/Ku80 heterodimer in eukaryotes. Ku acts as a scaffold protein to recruit other proteins involved in DSB repair, including nucleases to resect the damaged DNA, family B and family X DNAPs to fill the gap, and DNA ligase to ligate the DNA strands [251]. NHEJ is common in eukaryotes; the lack of requirement for a homologous partner means this is an effective method of DSB repair during the G1 phase of the cell cycle, when only a single genome copy is present.

Ku proteins are conserved in bacteria, yeast and higher eukaryotes, but to date only a few archaeal species have been shown to encode Ku proteins, which have most likely been acquired from bacteria by LGT [258,371]. To date, a complete NHEJ system has only been identified in a single archaeon, *Methanocella paludicola* [371]. Homologues of Ku are not found in *H. volcanii* [372,373]; instead an alternative mechanism of microhomology-mediated end joining (MMEJ) operates to repair DSBs [374,375].

#### 2.8.2. Microhomology-mediated end joining

While NHEJ does not require any homology, microhomology-mediated end joining (MMEJ) involves the annealing of short homologous sequences at broken DNA ends. The products of MMEJ are always associated with gene deletions and contribute to chromosome translocations and genome rearrangements. The basic mechanism for MMEJ involves resection of DNA ends, annealing of the micro-homologous region, the removal of the DNA flaps by the structure-specific nuclease Fen1, filling of the gap by DNA polymerases and ligation by DNA ligase [376–378] (figure 4*a* and table 1).

MMEJ has been observed directly in a small number of archaeal species, including *H. volcanii* and *S. islandicus* [374,375,379]. However, the detailed enzymology of MMEJ in archaea remains unknown. Due to the lack of signature proteins dedicated to MMEJ (in contrast with the requirement for Ku protein in NHEJ), the prevalence of MMEJ is unknown but likely to be widespread. *H. volcanii* uses MMEJ to repair DSBs; this process is stimulated (directly or indirectly) by the Rad50-Mre11 complex, which restrains HR and instead promotes MMEJ as the immediate pathway of DSB repair [374]. HR is later used to restore the repaired allele.

#### 2.8.3. Homologous recombination

The mechanism of HR is conserved throughout the three domains of life. HR is an essential process in archaea that provides a high-fidelity DSB repair mechanism, compared with other error-prone end-joining processes. This high fidelity is due to the use of homologous DNA duplex as the template for repair. Regulation of HR is essential to maintain genome integrity and avoid DNA rearrangements [380,381]. When a DSB occurs, it must be processed to generate ssDNA tails (pre-synapsis). This ssDNA tail then invades nearby duplex DNA with sequence homology, creating a D-loop (displacement loop) (synapsis). The final step of HR is post-synapsis, in which resolution of HR structures occurs, generating a crossover (where genetic exchange takes place) or a non-crossover (no genetic exchange) product (figure 4*b* and table 1).

HR is the best-studied DSB pathway in archaea [382]. Alongside its role in DSB repair, HR has been implicated in recombination-dependent replication (RDR), the restart of stalled DNA replication forks and in increasing genetic diversity [60,383,384]. The *H. volcanii* genome is highly polyploid with a genome copy number of approximately 20 copies per cell [12]; having multiple copies of DNA can be advantageous for efficient repair by HR as this increases the chances of having a non-damaged homologous template for DNA repair.

#### 2.8.4. Pre-synapsis

##### 2.8.4.1. Rad50-Mre11 complex

The Rad50-Mre11 complex is present in all domains of life [385,386]. In eukaryotes, Rad50-Mre11 is involved in the early steps of DSB repair, being one of the primary protein complexes recruited to the site of damage. Mre11 is an ATP-independent dsDNA exonuclease and ssDNA endonuclease, while Rad50 has ATP-dependent DNA-binding activity [387–391].

Rad50-Mre11 processes DSB ends by resecting the 5′ strand, generating a short single-stranded 3′ overhang. This overhang is then coated by a recombinase protein (RadA in archaea) to facilitate strand invasion [392,393]. In thermophilic archaea (e.g. *S. islandicus* and *T. kodakarensis*), Rad50 and Mre11 are essential for cell viability [111]. By contrast, in the halophile *Halobacterium* sp. NRC-1, Mre11 is essential while Rad50 is dispensable, indicating a Rad50-independent function of Mre11 [394]. In *H. volcanii*, deletions of *rad50* (HVO_0854) and *mre11* (HVO_0853) are viable and mutants show increased resistance to various types of DNA damage, including UV, ionizing radiation and MMS. However, these mutants recover slowly and exhibit higher rates of HR at DSBs than the wild type [374]. In a polyploid organism like *H. volcanii*, the action of Rad50-Mre11 in temporarily restraining HR may help prevent DNA ends from engaging with multiple homologous partners; after the number of available DNA ends has been reduced by MMEJ, DSBs are ultimately repaired by HR [374].

In addition, the *H. volcanii* Rad50-Mre11 complex has been shown to be involved in nucleoid compaction following genomic stress. Such nucleoid compaction is another mechanism to ensure DSB ends remain within close proximity [395], which may aid the search for intact DNA partners during HR and faster recruitment of DNA repair proteins to the sites of damage; similar mechanisms are seen in eukaryotes [391,396,397]. This compaction process is independent of the recombinase protein RadA (HVO_0104) [395], unlike in bacteria [398,399].

##### 2.8.4.2. HerA-NurA

In thermophilic archaea, *herA* and *nurA* are encoded in the same operon as *rad50* and *mre11* [400]. HerA and NurA have been shown to interact with each other both *in vitro* and *in vivo* [401]. The HerA-NurA ATP-dependent helicase-nuclease complex cooperates with Rad50-Mre11 to coordinate the repair of DSBs, although the mechanism has not yet been described in detail [400,402–404]. The ATPase activity of HerA, the nuclease activity of NurA and their interaction are essential for *Sulfolobus* viability [405]. HerA and NurA proteins are not present in *H. volcanii.* Bacterial homologues of HerA and NurA have been identified and were initially thought to be essential for cell viability [111]. Genetic studies have shown this is not the case in bacterial species *Deinococcus radiodurans* and *Thermus thermophilus* [406]; in the latter, deletion of *nurA* and *herA* has no effect on cell growth. Unexpectedly, *T. thermophilus* cells lacking *nurA* and *herA* show increased resistance to UV irradiation and MMC treatment [401].

#### 2.8.5. Synapsis

##### 2.8.5.1. Rada

The 3′ ssDNA tail generated during pre-synapsis acts to invade a homologous duplex; this is facilitated by coating of the ssDNA with a recombinase protein. The strand exchange protein, or recombinase, promotes homology search and catalyses strand invasion, giving rise to a D-loop (displacement loop) [381,407]. Archaea contain homologues of eukaryotic HR proteins, including the evolutionarily conserved RecA-family strand exchange protein, called RadA in archaea, RecA in bacteria and Rad51 in eukaryotes.

Archaeal RadA is more similar to eukaryotic Rad51 than to bacterial RecA [408]. Like bacterial cells lacking RecA, RadA-deficient *H. volcanii* cells show growth defects, a lack of homologous recombination, and increased sensitivity to DNA damage agents [409–412], indicating an important role for HR in archaea. In thermophilic archaea, RadA is essential [111], suggesting these organisms are critically reliant on HR for repair and/or replication.

##### 2.8.5.2. RadB

Paralogues of RecA-family proteins are present in many organisms where they generally function as recombination mediators [413–418]. Eukaryotic recombination mediators, such as BRCA2 in humans, and Rad52 and Rad55–Rad57 in yeast, act to displace RPA and facilitate the loading of recombinase Rad51 onto DNA [419,420]. Rad55–Rad57 are paralogues of Rad51 and have been shown to stabilize Rad51 filament formation [421]; BRCA2 and Rad52, while recombination mediators, are not paralogues of Rad51.

In archaea, RadB is a paralogue of RadA that is present only in Euryarchaea [422]. In *H. volcanii,* RadB (HVO_2383) acts as a recombination mediator that interacts with RadA and promotes its polymerization on ssDNA [412]. Strains in which *radB* has been deleted show a phenotype similar to cells lacking *radA*; defects include growth retardation, low levels of recombination and DNA damage sensitivity [411,412]. Two suppressor mutations that alleviate the *ΔradB* phenotype have been identified in *H. volcanii* RadA, S101P and A196 V, which suggest that RadB induces a conformational change in RadA, promoting its polymerization on ssDNA [412].

#### 2.8.6. Post-synapsis

##### 2.8.6.1. Hjc

During HR, four-way DNA intermediates (Holliday junctions; HJs) are formed by strand exchange between homologous DNA molecules. Resolvases, a group of highly specialized structure-specific metal-dependent endonucleases catalyse the cleavage of HJs into two DNA duplexes [423]. Resolvases are ubiquitous and are found in bacteria, eukaryotes, archaea and even in some viruses, although they are not directly related [424,425].

In *E. coli*, HJs are resolved by the RuvABC complex. The initial RuvAB complex, comprising a RuvA tetramer and two RuvB hexamers, specifically binds the HJ and promotes branch migration, following which RuvC cleaves the junction [426]. Eukaryotic HJ resolution is more complex than the bacterial counterpart and multiple endonucleases have been implicated in the resolution of HJs, including Yen1 (GEN1), Mus81-Mms4 (Eme1), Slx1-Slx4 and XPF-ERCC1 [427,428].

In archaea, the Hjc resolvase has been implicated in the cleavage of HJs. Hjc is present in all archaeal species and has been shown to interact with various proteins involved in DNA replication and repair, including the helicase Hel308 [429], clamp protein PCNA [430] and ATPase HerA [431]. Some *Sulfolobus* species contain an additional resolvase Hje, which shows higher DNA cleavage activity than Hjc [431,432]. *H. volcanii* Hjc (HVO_0170) is non-essential, has been shown to act in the same pathway as RadA and is necessary for efficient growth in the presence of cross-linking agent MMC [219].

In *S. islandicus*, Hjc is regulated by phosphorylation, which acts to inhibit its catalytic activity [433]. Interestingly, Hjc phosphomimetic mutants of *S. islandicus* exhibit increased resistance to DNA damaging agents, suggesting that phosphorylation acts to redirect repair to avoid HR that is dependent on Hjc [433]. While the serine residues targeted for phosphorylation in *S. islandicus* Hjc are not conserved in other archaeal species, modification of Hjc and other HR proteins by phosphorylation in *S. acidocaldarius* is well documented [434]*.* This regulatory mechanism could have evolved early during evolution, as the activity of several HJ resolvases is also regulated by phosphorylation in eukaryotes [435–437]. However, it remains unknown whether such post-translational modifications are used to regulate Hjc in *H. volcanii*.

##### 2.8.6.2. Hef

Hef (helicase-associated endonuclease fork-structure DNA) is a member of the XPF family of structure-specific endonucleases known to act on branched, flapped and forked DNA structures [438]. Hef features two distinct domains: an N-terminal helicase domain and C-terminal endonuclease domain [438–441]. All archaea encode an XPF family endonuclease, but Hef is specific to euryarchaeal species.

In *H. volcanii*, Hef (HVO_3010) is not involved in NER and instead a bacterial Uvr system is used [219]. However, Hef has been implicated in NER in other species [111]. Both *H. volcanii* and *T. kodakarensis* mutants deleted for *hef* show sensitivity to MMC [111,307]. In *H. volcanii*, Hef is non-essential but cannot be deleted from cells lacking Hjc, suggesting these two proteins participate in alternative mechanisms for the resolution of recombination intermediates [219]. Redundancy of proteins involved in HJ resolution has also been described for other species of archaea; for example, HJ resolvases Hje and Hjc in *S. islandicus* are redundant [431]. In *H. volcanii*, Hef is required for cell viability in the absence of HR and is recruited to sites of DNA replication fork arrest [219,442]. This indicates a key role for Hef in the restart of stalled replication forks by RDR. Accordingly, Hef has been shown to interact with PCNA in *T. kodakarensis* [443], *P. abysii* [444] and *T. acidophilum* [445]. This interaction likely ensures availability of Hef at the replication fork, facilitating its role in RDR. While an interaction with PCNA has not yet been shown in *H. volcanii*, Hef in this species features a PIP box, indicating that Hef : PCNA interactions are common to most euryarchaea.

Alongside Hef, archaea from the phylum Euryarchaea encode a conserved protein HAN (Hef-associated nuclease), a RecJ-like protein displaying 3′–5′ exonuclease activity [443]. Hef and HAN interact with PCNA in *T. kodakarensis*, although Hef cannot bind both PCNA and HAN simultaneously [443]. Deletion of *han* in *H. volcanii* (HVO_1018; also called *recJ3*) results in increased sensitivity to DNA damage agent MMS, but not to other genotoxic agents such as H_2_O_2_, 4NQO or MMC [446]. It has been proposed that HAN acts to coordinate the helicase and nuclease activities of Hef during the processing of stalled replication forks [110,442].

##### 2.8.6.3. Hel308

Hel308 is a Ski2 family 3′–5′ helicase found in archaea and metazoans (where it is named HelQ/PolQ) [447–450]; however, Hel308 is absent from fungi and bacteria. Hel308 is able to unwind various dsDNA structures *in vitro*, showing the preference for forked DNA and D-loops, and can remove bound proteins during translocation; these activities suggest a role in HR and the restart of stalled replication forks [411]. Hel308 has been shown to interact with other proteins involved in HR, including the BCDX2-Rad51 paralogue complex in humans [450,451], Hjc resolvase in *S. tokodaii* [429] and RPA in *M. thermautotrophicus* [452]. Hel308 has been proposed to act at blocked DNA replication forks, whereby it unwinds the parental strands and facilitates the loading of other factors required for the restart of the stalled replication fork.

Hel308 is not essential in the majority of archaeal species that encode it, with the exception of *S. tokodaii* and *S. islandicus* [429,453]. Mutants of Hel308 in *H. volcanii* (HVO_0014) are viable but slow-growing and show sensitivity to DNA cross-linking agents such as MMC (T.A. 2020*,* unpublished data); a similar phenotype has been seen in human *ΔhelQ* mutants [450]. This implicates *H. volcanii* Hel308 in the processing and repair of inter-strand cross-linked DNA lesions.

### 2.9. Recombination-dependent DNA replication

Replication origins were originally thought to be indispensable for cellular life, and deletion of origins was found to lead to impaired growth or cell death [454]. Work in bacterial model species *E. coli* showed that in the absence of origins, DNA replication can be primed from D-loops or R-loops (RNA displacement loops); these structures undergo remodelling to form a canonical replication fork [455]. For utilization of an R-loop, the invading RNA strand must remain intact and since RNase H proteins usually act to degrade RNA : DNA hybrids, RNase H gene(s) must be inactivated to allow R-loop-mediated replication to occur [454,455]. Both DNA- and RNA-dependent mechanisms of originless replication have been shown to be reliant on bacterial recombinase, RecA, indicating that the process uses HR; hence, it is known asRDR [456].

Surprisingly, it is possible to delete all chromosomal origins of replication in *H. volcanii* without affecting viability; in fact, strains deleted for origins grow 7.5% faster than wild type [60]. As in *E. coli,* cells deleted for replication origins become dependent upon the recombinase protein (here RadA), indicating that originless strains use RDR and hence HR becomes essential [60]. Marker frequency analysis (MFA) in originless *H. volcanii* shows a flat replication profile, indicating a lack of specific initiation and termination sites [60]. Similarly, the euryarchaeon *T. kodakarensis* has been shown to be capable of DNA replication in the absence of origins, again using a mechanism dependent on HR [457]. As in *H. volcanii*, MFA analysis of *T. kodakarensis* shows a flat replication profile in originless strains. But unlike *H. volcanii*, a flat replication profile is also seen in wild-type *T. kodakarensis*, suggesting that the origin, while present, is not used under laboratory conditions [457].

Both *H. volcanii* and *T. kodakarensis* are highly polyploid species. Having a large number of genome copies increases the chance of finding a homologous DNA duplex to invade and carry out HR; this may explain why RDR can occur in these organisms with relative ease. Similar findings have been made with polyploid species of cyanobacteria, which are able to carry out efficient DNA replication in the absence of the initiator protein DnaA [458]. Such a link between high ploidy and a capacity for RDR may explain why organisms with low ploidy, including *E. coli* and higher eukaryotes, are reliant upon origins to replicate their DNA; the lack of homologous DNA sequences would restrict the potential for RDR. Within archaea, members of the crenarchaea have been shown to contain only 1–2 genome copies and, unlike polyploid euryarchaea, have a defined cell cycle [459,460]. When ploidy is reduced to a single copy at the G1 stage of the cell cycle, RDR is no longer possible. This has been shown for *S. islandicus*, where one of its three replication origins must be maintained for cell viability [56].

However, not all polyploid Euryarchaea are capable of originless replication, including two close relatives of *H. volcanii*: *Haloarcula hispanica* and *Haloferax mediterranei*. The two replication origins of *H. hispanica* cannot be deleted at the same time, indicating that at least one origin is essential for viability [461]. In *H. mediterranei*, deletion of all three chromosomal origins is possible but this leads to the activation of a dormant origin [462]; the dormant origin appears to have been acquired by LGT and is not found in *H. volcanii*. The variety of responses to origin deletion in archaea hints at complex differences between species in their capacity for RDR, and further work is needed to elucidate the specific mechanisms involved.

## 3. Conclusion

Over the past three decades, the development and application of genetic and biochemical tools for *H. volcanii* have accelerated. These tools have, in turn, increased our understanding of the mechanisms of DNA replication and repair, both in this organism, in halophiles and in archaea generally. The increasing number of model archaeal species has provided additional insights into diversity across the domain, where species within a phylum may use radically different mechanisms for basic processes such as DNA replication.

The identification and study of archaeal DNA repair and replication enzymes has provided invaluable information on their eukaryotic counterparts. On the other hand, the development of tools specific for archaea has shed light on adaptations and proteins specific to this domain. Such a two-pronged approach—to determine what is common with eukaryotes and what is unique to archaea—is needed to uncover the evolutionary history of DNA replication and repair.

The ease of genetics and availability of DNA damaging agents have allowed the identification of key DNA repair enzymes in *H. volcanii*. The pathways used are counterparts of either entire eukaryotic or bacterial systems, with limited substitution within a pathway. Such preservation of pathway integrity is explained by the complexity hypothesis [463], which states that genes encoding proteins that function in complexes (e.g. DNA repair) are less frequently transferred via LGT and then only as entire operons. By contrast, the genes encoding proteins that do not form complexes (e.g. those that act in central metabolism) can be transferred successfully by LGT. In the case of *H. volcanii*, LGT has led to wholesale displacements (e.g. bacterial UvrABC has displaced the archaeal NER pathway), alternative activities that are used interchangeably (e.g. archaeal LigA and bacterial LigN are both active as DNA ligases) or acquisitions that are not used (e.g. bacterial DnaG is not used as primase, instead archaeal PriS/L plays that role). These three scenarios illustrate how *H. volcanii* is not just a good model organism for the study of DNA replication and repair mechanisms, but is also invaluable for the study of LGT and evolution.

While our understanding of DNA replication and repair in *H. volcanii* has undoubtedly increased, numerous questions remain. The ever-increased repertoire of genetic and biochemical tools for *H. volcanii* cements its place as a *bona fide* archaeal model organism. These tools will prove invaluable to answer open questions and thereby increase our understanding of DNA replication and repair in archaea.

## Data accessibility

This article has no additional data.
